# Circulating adrenomedullin estimates survival and reversibility of organ failure in sepsis: the prospective observational multinational Adrenomedullin and Outcome in Sepsis and Septic Shock-1 (AdrenOSS-1) study

**DOI:** 10.1186/s13054-018-2243-2

**Published:** 2018-12-21

**Authors:** Alexandre Mebazaa, Christopher Geven, Alexa Hollinger, Xavier Wittebole, Benjamin Glen Chousterman, Alice Blet, Etienne Gayat, Oliver Hartmann, Paul Scigalla, Joachim Struck, Andreas Bergmann, Massimo Antonelli, Albertus Beishuizen, Jean-Michel Constantin, Charles Damoisel, Nicolas Deye, Salvatore Di Somma, Thierry Dugernier, Bruno François, Stephane Gaudry, Vincent Huberlant, Jean-Baptiste Lascarrou, Gernot Marx, Emmanuelle Mercier, Haikel Oueslati, Peter Pickkers, Romain Sonneville, Matthieu Legrand, Pierre-François Laterre, AdrenOSS-1 study investigators, Pierre François Laterre, Caroline Berghe, Marie-France Dujardin, Suzanne Renard, Xavier Wittebole, Christine Collienne, Diego Castanares Zapatero, Thierry Dugernier, Marco Vinetti, Nicolas De Schryver, Anne Thirifays, Jacques Mairesse, Vincent Huberlant, Hélène Petre, Isabelle Buelens, Pierre Henin, Hugues Trine, Yves Laurent, Loix Sébastien, Paul Geukens, Laurent Kehl, Bruno François, Philippe Vignon, Nicolas Pichon, Emmanuelle Begot, Anne-Laure Fedou, Catherine Chapellas, Antoine Galy, Nicolas Rodier, Ludmilla Baudrillart, Michelle Nouaille, Séverine Laleu, Claire Mancia, Thomas Daix, Paul Bourzeix, Isabelle Herafa, Anne-Aurore Duchambon, Jean Baptiste Lascarrou, Maud Fiancette, Gwenhael Colin, Matthieu Henry-Lagarrigue, Jean-Claude Lacherade, Christine Lebert, Laurent Martin-Levèvre, Isabelle Vinatier, Aihem Yehia, Konstantinos Bachoumas, Aurélie Joret, Jean Reignier, Cécille Rousseau, Natacha Maquigneau, Yolaine Alcourt, Vanessa Erragne Zinzonni, Angélique Deschamps, Angelina Robert, Emmanuelle Mercier, Véronique Simeon-Vieules, Aurélie Aubrey, Christine Mabilat, Denis Garot, Stephan Ehrmann, Annick Legras, Manikikian, Youenn Jouan, Pierre François Dequin, Antoine Guillon, Laetitia Bodet-Contentin, Emmannuelle Rouve, Charlotte Salmon, Lysiane Brick, Stéphanie Massat, Arnaud Desachy, Marie Anne Fally, Laurence Robin, Christophe Cracco, Charles Lafon, Sylvie Calvat, Stéphane Rouleau, David Schnell, Sigismond Lasocki, Philippe Fesard, Damien Leblanc, Guillaume Bouhours, Claire Chassier, Mathieu Conte, Thomas Gaillard, Floriane Denou, Mathieu Kerymel, Marion Guyon, Anthéa Loiez, Stéphanie Lebreton, Ferhat Meziani, Hayat Allam, Samir Chenaf, Hassène Rahmani, Sarah Heenen, Christine Kummerlen, Xavier Delabranche, Alexandra Boivin, Raphaël Clere-Jehl, Yannick Rabouël, Julien Pottecher, Sophie Bayer, Catherine Metzger, Stéphane Hecketsweiler, Pierre Olivier Ludes, Hortense Besancenot, Nadia Dhif, Guy Freys, Jean-Marc Lessinger, Anne Launoy, Aude Ruimy, Alain Meyer, M. Szozot, Alexandre Mebazaa, Nicolas Deye, Etienne Gayat, Marie-Céline Fournier, Sarra Abroug, Badr Louadah, Elodie Feliot, Sebastian Voicu, I. Malissin, Bruno Megarbane, Philippe Manivet, Gardianot Victori, Da Silva Kelly, Béatrice La Foucher, Valérie Pierre, Lamia Kerdjana, Thomas Beeken, Antoine Goury, Pierre Garcon, Samuel Gaugain, Benjamin Glen Chousterman, Benjamin Huot, Romain Barthelemy, Benjamin Soyer, Laurent Jacob, Matthieu Legrand, Marie-Céline Fournier, Francine Bonnet, Chloé Legall, Haikel Oueslati, Alexandru Cupaciu, Philippe Manivet, Badr Louadah, Romain Sonneville, Sophie Letrou, Lila Bouadma, Bruno Mourvillier, Véronique Deiler, Eric Magalhaes, Mathilde Neuville, Jean-François Timsit, Aguila Radjou, Stéphane Gaudry, Emeline Dubief, Jonathan Messika, Béatrice La Combe, Damien Roux, Guillaume Berquier, Mohamed Laissi, Jean-Damien Ricard, Jean-Michel Constantin, Sebastien Perbet, Julie Delmas, Julien Pascal, Sophie Cayot, Renaud Guerin, Matthieu Jabaudon, Laurence Roszyk, Christine Rolhion, Justine Bourdier, Mathilde Lematte, Charlène Gouhier, Camille Verlhac, Thomas Godet, Sophiano Radji, Elodie Caumon, Sandrine Thibault, Nikolaus Marx, Tobias Schuerholz, Jessica Pezechk, Florian Feld, Christian Brülls, Thorben Beeker, Tim-Philipp Simon, Robert Deisz, Achim Schindler, Bianca Meier, Thorsten Janisch, Andreas Hohn, Dirk Schedler, Wolfgang Wetsch, Daniel Schröder, Andreas Meier-Hellmann, Alexander Lucht, Robert Henker, Magdalena Römmer, Torsten Meinig, Kai D. Zacharowski, Patrick Meybohm, Simone Lindau, Haitham Mutlak, Stefan Kluge, Grit Ringeis, Birgit Füllekrug, Brigitte Singer, Axel Nierhaus, Katrin Bangert, Geraldine de Heer, Daniel Frings, Valentin Fuhrmann, Jakob Müller, Jörg Schreiber, Barbara Sensen, Stephanie Siedler, Annekatrin Siewecke, Gerold Söffker, Dominic Wichmann, Mélanie Kerinn, Ulrich Jaschinski, Ilse Kreuser, Marlene Zanquila, Andreas Kortgen, Frank Bloos, Falk Gonnert, Daniel Thomas-Rüddel, Anja Haucke, Steffi Kolanos, Karina Knuhr Kohlberg, Petra Bloos, Katrin Schwope, Salvatore Di Somma, Marino Rossella, Veronica Russo, Santarelli Simona, Christopher Bartoli, Sylvia Navarin, Cristina Bongiovanni, Michela Orru, Daniela Quatrocchi, Giada Zoccoli, Antonella Varchetta, Massimo Antonelli, Gennaro de Pascale, Maria Sole Vallecoccia, Salvatore Lucio Cutuli, Valentina Digravio, Daniela Quattrochi, Sonia D’Arrigo, Filippo Elvino Leone, Bert Beishuizen, Martin Rinket, Natalie Border, Mariska Bos-Burgmeijer, Astrid Braad, S. Papendorp, Alexander Cornet, J. Vermeijden, Ronald J. Trof, Peter Pickkers, Marieke van de A, Helen Van Wezel, Leo Heunks, Natalie Border, Chantal Luijten-Arts, Astrid Hoedemaekers, Hans van der Hoeven, Noortje Roovers, Pleun Hemelaar

**Affiliations:** 1 Department of Anesthesiology, Burn and Critical Care Medicine, AP-HP, Saint Louis and Lariboisière University Hospitals, 2 rue A. Paré, 75010 Paris, France; 2 Inserm 942, Paris, France; 3 University Paris Diderot, Paris, France; 4 Department of Intensive Care Medicine, Radboud University Medical Center, Geert Grooteplein Zuid 10, 6500 HB Nijmegen, The Netherlands; 5 Department of Anesthesia, Surgical Intensive Care, Prehospital Emergency Medicine and Pain Therapy, University Hospital Basel, Basel, Switzerland; 6 Department of Critical Care Medicine, St Luc University Hospital, Université Catholique de Louvain, Brussels, Belgium; 7 sphingotec GmbH, Hennigsdorf, Germany; 8 Adrenomed AG, Hennigsdorf, Germany; 9 Fondazione Policlinico Universitario A. Gemelli, Rome, Italy; 10 Department of Intensive Care, Medische Spectrum Twente, Enschede, The Netherlands; 11 Department of Perioperative Medicine, University Hospital of Clermont-Ferrand, Clermont-Ferrand, France; 12 Sant’ Andrea Hospital, Rome, Italy; 13 Clinique St Pierre, Ottignies, Belgium; 14 ICU Department, CHU Dupuytren, Limoges, France; 15 INSERM CIC 1435/UMR 1092, Limoges, France; 16 Hôpital Louis Mourier, Colombes, France; 17 Hôpital Jolimont, Haine-St-Paul, Belgium; 18 Centre Hospitalier Universitaire de Nantes, Nantes, France; 19 Klinik für Operative Intensivmedizin und Intermediate Care, Universitätsklinikum der RWTH, Aachen, Germany; 20 CHU de Tours, Tours, France; 21 Hopital Bichat Claude-Bernard, Paris, France; 22 Department of Critical Care Medicine, Saint Luc University Hospital, Université Catholique de Louvain, Avenue Hippocrate 10, 1200 Brussels, Belgium

**Keywords:** Biomarker, Outcome, Sepsis-2, Sepsis-3

## Abstract

**Background:**

Adrenomedullin (ADM) regulates vascular tone and endothelial permeability during sepsis. Levels of circulating biologically active ADM (bio-ADM) show an inverse relationship with blood pressure and a direct relationship with vasopressor requirement. In the present prospective observational multinational Adrenomedullin and Outcome in Sepsis and Septic Shock 1 (, AdrenOSS-1) study, we assessed relationships between circulating bio-ADM during the initial intensive care unit (ICU) stay and short-term outcome in order to eventually design a biomarker-guided randomized controlled trial.

**Methods:**

AdrenOSS-1 was a prospective observational multinational study. The primary outcome was 28-day mortality. Secondary outcomes included organ failure as defined by Sequential Organ Failure Assessment (SOFA) score, organ support with focus on vasopressor/inotropic use, and need for renal replacement therapy. AdrenOSS-1 included 583 patients admitted to the ICU with sepsis or septic shock.

**Results:**

Circulating bio-ADM levels were measured upon admission and at day 2. Median bio-ADM concentration upon admission was 80.5 pg/ml [IQR 41.5–148.1 pg/ml]. Initial SOFA score was 7 [IQR 5–10], and 28-day mortality was 22%. We found marked associations between bio-ADM upon admission and 28-day mortality (unadjusted standardized HR 2.3 [CI 1.9–2.9]; adjusted HR 1.6 [CI 1.1–2.5]) and between bio-ADM levels and SOFA score (*p* < 0.0001). Need of vasopressor/inotrope, renal replacement therapy, and positive fluid balance were more prevalent in patients with a bio-ADM > 70 pg/ml upon admission than in those with bio-ADM ≤ 70 pg/ml. In patients with bio-ADM > 70 pg/ml upon admission, decrease in bio-ADM below 70 pg/ml at day 2 was associated with recovery of organ function at day 7 and better 28-day outcome (9.5% mortality). By contrast, persistently elevated bio-ADM at day 2 was associated with prolonged organ dysfunction and high 28-day mortality (38.1% mortality, HR 4.9, 95% CI 2.5–9.8).

**Conclusions:**

AdrenOSS-1 shows that early levels and rapid changes in bio-ADM estimate short-term outcome in sepsis and septic shock. These data are the backbone of the design of the biomarker-guided AdrenOSS-2 trial.

**Trial registration:**

ClinicalTrials.gov, NCT02393781. Registered on March 19, 2015.

**Electronic supplementary material:**

The online version of this article (10.1186/s13054-018-2243-2) contains supplementary material, which is available to authorized users.

## Introduction

Adrenomedullin (ADM) is a free circulating peptide with potent vascular properties, including benefits for endothelial barriers at physiological levels. ADM has previously been described as a “double-edged sword” in sepsis [1] because high levels of ADM induce vasodilation and hypotension [2–4] on one hand while reinforcing the endothelial barrier and improving outcome on the other [5–10]. The potential of ADM as a prognostic biomarker has previously been studied in critically ill patients, often by measuring the inactive midregional pro-ADM [11, 12], or recently by direct measurement of the bioactive form of ADM (bio-ADM) [13, 14]. It has been shown repeatedly that bio-ADM greater than 70 pg/ml is associated with worse outcome [13, 14].

On the basis of previous results, we tested the hypothesis that modulating the ADM pathway in patients with high levels of circulating bio-ADM may improve short-term outcome in sepsis. Adrecizumab, a monoclonal anti-ADM antibody, has been shown to improve organ function in preclinical settings [15]. In order to design a human trial in which we would administer adrecizumab based on levels of bio-ADM, we needed to assess the relationship between initial levels of bio-ADM and short-term outcome in sepsis and in septic shock patients.

In the Adrenomedullin and Outcome in Sepsis and Septic Shock 1 (AdrenOSS-1) study, we investigated whether the initial plasma concentration of bio-ADM (on intensive care unit [ICU] admission and after 48 h) may provide insight into 28-day survival and the recovery of organ function.

## Methods

### Study design

AdrenOSS-1 was a European prospective observational study. Twenty-four centers in five countries (France, Belgium, The Netherlands, Italy, and Germany) contributed to the trial achievement of 583 enrolled patients. Patients were recruited from June 2015 to May 2016. The study protocol was approved by the local ethics committees and was conducted in accordance with Directive 2001/20/EC, as well as good clinical practice (International Conference on Harmonization Harmonized Tripartite Guideline version 4 of May 1, 1996, and decision of November 24, 2006) and the Declaration of Helsinki.

The study enrolled patients aged 18 years and older who were (1) admitted to the ICU for sepsis or septic shock or (2) transferred from another ICU in the state of sepsis and septic shock within less than 24 h after admission. Included patients were stratified by severe sepsis and septic shock based on definitions for sepsis and organ failure from 2001 [16]. In the present article, the term “sepsis” refers to the updated definition of Sepsis-3 [17]. Concerning septic shock, most data presented in this article are based on the former definition [16], except for the confirmatory analyses presented in the last paragraph of the “Results” section, for which the new Sepsis-3 definition of septic shock was used [17].

Exclusion criteria were pregnancy, vegetative coma, and participation in an interventional trial in the preceding month. Informed consent was obtained from all patients or their lawful representatives prior to enrollment in the study. Patients were treated according to local practice, and treatments as well as procedures were registered.

The primary endpoint was 28-day mortality. Secondary endpoints concerned organ failure (as defined by the Sequential Organ Failure Assessment [SOFA] score) and organ support, vasopressor/inotrope use, fluid balance, and use of renal replacement therapy (RRT), as well as validation of the previously identified cutoff value of 70 pg/ml [14]. The latter was identified as the optimal screening cutoff for AdrenOSS-2, an ongoing proof-of-concept and dose-finding phase II trial assessing adrecizumab (an antibody modulating circulating bio-ADM) in patients with early septic shock (NCT03085758). The relationship between cardiovascular SOFA subscore and bio-ADM, being a biomarker of vascular dysfunction, was evaluated.

### Collection of patient data

Upon admission, demographics (age, sex), body mass index, presence of septic shock, type of ICU admission, organ dysfunction scores (SOFA, Acute Physiologic Assessment and Chronic Health Evaluation II [APACHE II]), origin of sepsis, preexisting comorbidities (i.e., treated within the last year), past medical history, laboratory values, and organ support were recorded, and blood was drawn for measurement of bio-ADM and other markers.

After patient enrollment, the following data were collected daily during the first week: SOFA score, antimicrobial therapies, fluid balance, ventilation status, Glasgow Coma Scale score, central venous pressure, need for RRT, invasive procedures for sepsis control, and vasopressor/inotrope treatment. Moreover, discharge status and mortality were recorded on day 28 after ICU admission.

### Sample collection

Blood for the central laboratory was sampled within 24 h after ICU admission and on day 2 (mean 47 h, SD 9 h) after the first sample. Samples were subsequently processed and stored at − 80 °C before transfer to the central laboratory for blinded bio-ADM analysis organized by the study sponsor (sphingotec GmbH, Hennigsdorf, Germany). Routine analyses (e.g., partial pressure of arterial oxygen, lactate) were performed by the local laboratories.

### Bio-ADM measurement

Bio-ADM was measured using a recently developed immunoassay provided by sphingotec GmbH. For details and design principles on the assay, *see* publications by Marino et al. [14] and Weber et al. [18]. The analytical assay sensitivity was 2 pg/ml.

### Statistical analyses

Results are presented as number and percentage, mean and SD, or median and IQR, depending on their distribution. Group comparisons for continuous variables were performed using the Kruskal-Wallis test, and appropriate post hoc tests were applied if necessary. Categorical data were compared using the chi-square test with simulated *p* values using 2000 replicates. Biomarker data were log-transformed if necessary. Cox proportional hazards regression was used to analyze the effect of risk factors on survival in uni- and multivariable analyses. The assumptions of proportional hazards were tested for all variables. For continuous variables, HRs were standardized to describe the HR for a biomarker change of one IQR. CIs (95% CI) for risk factors and significance levels for chi-square (Wald) test are given. The predictive value of each model was assessed by the model likelihood ratio chi-square statistic. The concordance index (C index) is given as an effect measure. It is equivalent to the concept of AUC adopted for binary outcome. For multivariable models, a bootstrap-corrected version of the C index is given. To test for added predictive value, we used the likelihood ratio chi-square test for nested models to assess whether bio-ADM adds predictive value to a clinical model or a risk score. Survival curves plotted by the Kaplan-Meier method using quartiles or predefined cut points (70 pg/ml) of bio-ADM were used for illustrative purposes. ROC curve analysis was applied for 28-day mortality to determine the optimal Youden cutoff in this cohort.

A two-sided *p* value of 0.05 was considered statistically significant. All analyses were performed using R version 2.5.1 (http://www.r-project.org, library Design, Hmisc, ROCR) and IBM SPSS Statistics version 22.0 software (IBM, Armonk, NY, USA).

## Results

A total of 583 patients were included in the AdrenOSS-1 study. Patient characteristics, organ dysfunction scores, physiological and laboratory values, organ support upon admission, and outcome parameters are presented in Table 1. The median bio-ADM level at admission was 80.5 pg/ml [IQR 41.6–148.1] in our studied patients; 55.9% had bio-ADM level greater than 70 pg/ml at admission, and 44.1% had a bio-ADM less than 70 pg/ml. Of note, patients with septic shock had a significantly higher bio-ADM concentration at admission than patients with sepsis (114.4 [62.6–214.5] versus 57.5 pg/ml [31.2–101.5], *p* < 0.0001).

**Table 1:** Patient characteristics

Patient characteristics	All	Bio-ADM < 70 pg/ml at admission	Bio-ADM > 70 pg/ml at admission	*p* Value*	No.
Epidemiological data	*n* = 583	*n* = 257	*n* = 326		
Bio-ADM at admission (pg/ml)	80.5 [41.5–148.0]	36.9 [27.1–51.0]	136.7 [97.6–241.0]	< 0.0001	
Age (years)	66 [55–76]	64 [53–75]	67 [58–76]	0.0052	
Male sex (n, %)	364 (62.4)	171 (66.5)	193 (59.2)	0.0837	
Body mass index (kg/m^2^)	25.7 [22.9–30.1]	25.0 [22.3–28.4]	26.7 [23.2–31.6]	0.0013	
Septic shock at admission	293 (50.3)	84 (32.7)	209 (64.1)	< 0.0001	
Type of ICU admission				< 0.0001	
Medical	473 (81.1)	230 (89.5)	243 (74.5)		
Surgical - emergency procedure	93 (16)	21 (8.2)	72 (22.1)		
Surgical - elective procedure	17 (2.9)	6 (2.3)	11 (3.4)		
Origin of sepsis				< 0.0001	
Lung	218 (37.4)	129 (50.2)	89 (27.3)		
Bloodstream	90 (15.4)	31 (12.1)	59 (18.1)		
Urinary tract	62 (10.6)	10 (3.9)	52 (16)		
Catheter	29 (5)	9 (3.5)	20 (6.1)		
Peritonitis	31 (5.3)	12 (4.7)	19 (5.8)		
Endocarditis	31 (5.3)	12 (4.7)	19 (5.8)		
Bile duct infection	8 (1.4)	2 (0.8)	6 (1.8)		
CNS	4 (0.7)	4 (1.6)	0 (0)		
Skin and soft tissue	10 (1.7)	9 (3.5)	1 (0.3)		
Gynecologic	2 (0.3)	1 (0.4)	1 (0.3)		
Other	98 (16.8)	38 (14.8)	60 (18.4)		
Medical history^a^					
Any cardiac comorbidity	400 (68.6)	147 (57.2)	253 (77.6)	< 0.0001	
Chronic heart failure	60 (10.3)	19 (7.4)	41 (12.6)	0.0544	
Hypertension	293 (50.3)	105 (40.9)	188 (57.7)	< 0.0001	
Diabetes mellitus	160 (27.4)	57 (22.2)	103 (31.6)	0.0150	
Any noncardiac comorbidity	414 (71)	167 (65)	247 (75.8)	0.0058	
Chronic renal disease	76 (13.0)	19 (7.4)	57 (17.5)	0.0004	
Active/recent malignant tumors	124 (21.3)	34 (13.2)	90 (27.6)	< 0.0001	
Smoking (active)	117 (20.1)	63 (24.5)	54 (16.6)	0.0302	
COPD	89 (15.3)	37 (14.4)	52 (16.0)	0.6421	
Any chronic medication	371 (63.6)	138 (53.7)	233 (71.5)	< 0.0001	
Immunosuppressive therapy	46 (7.9)	11 (4.3)	35 (10.7)	0.0066	
Physiological values at admission					
Temperature (°C)	37.2 [36.4–38.2]	37.4 [36.6–38.2]	37.1 [36.2–38.1]	0.0034	
Mean blood pressure (mmHg)	75 [64–90]	81 [69–95]	72 [60–85]	< 0.0001	
Heart rate (beats/min)	104 [90–119]	100 [86–116]	105 [94–121]	0.0013	
Central venous pressure (mmHg)	8 [5–13]	8 [5–13]	10 [6–14]	0.2419	
Glasgow Coma Scale score	15 [14–15]	15 [14–15]	15 [14–15]	0.8161	
Fluid balance (ml)	1928 [592–3552]	1425 [500–2699]	2311 [764–4202]	< 0.0001	
Urine output for 24 h (ml)	1000 [450–1900]	1276 [650–2050]	800 [300–1650]	< 0.0001	
PaO_2_/FiO_2_	228 [137–340]	233.5 [140–360]	223 [137–337]	0.4995	
Laboratory values at admission					
Lactate (mmol/L)	1.4 [1.0–2.2]	1.1 [0.8–1.6]	1.8 [1.2–2.7]	< 0.0001	*n* = 562
Arterial pH	7.38 [7.3–7.44]	7.42 [7.36–7.46]	7.36 [7.27–7.42]	< 0.0001	
Bilirubin (μmol/L)	11 [6–19]	10 [6.5–17]	12 [6–21]	0.1360	
Platelets (10^9^/L)	190 [121–275]	196 [136–279]	181 [104–271]	0.0583	
Creatinine (mg/dl)	1.4 [0.9–2.2]	1 [0.7–1.4]	1.8 [1.2–2.9]	< 0.0001	
BUN or urea (mg/dl)	61 [37–107]	44 [28–69]	80 [50–127]	< 0.0001	
Hematocrit (%)	34 [29–38]	35 [30–38]	34 [29–38]	0.1010	
White blood cell count (per mm^3^)	12,525 [7200–18,585]	13,000 [8475–18,075]	12,025 [5942–19,025]	0.0547	
Troponin T, maximum on day 1	42 [18–158]	29 [14–124]	55 [25–176]	0.0230	*n* = 153
Troponin I, maximum on day 1	69 [20–246]	40 [11–228]	99 [40–289]	0.0049	*n* = 186
PCT, maximum on day 1 (ng/ml)	11.4 [1.9–49.8]	3.9 [0.9–19.5]	24 [6–84]	< 0.0001	*n* = 330
PCT, central laboratory (ng/ml)	10.2 [2.3–34.3]	3.7 [0.8–13.0]	18.2 [6.0–52.7]	< 0.0001	*n* = 583
BNP, maximum on day 1	257 [102–723]	187 [61–388]	473 [147–1154]	0.0004	*n* = 131
NT-proBNP, maximum on day 1	4382 [1525–11,565]	2170 [497–6633]	6116 [2816–15,431]	0.0001	*n* = 117
Organ support at admission					
Mechanical ventilation				0.0739	
Invasive	219 (37.6)	85 (33.1)	134 (41.1)		
Noninvasive	131 (22.5)	67 (26.1)	64 (19.6)		
None	233 (40.0)	105 (40.9)	128 (39.3)		
Renal replacement therapy	49 (8.4)	8 (3.1)	41 (12.6)	0.0001	
Vasopressors/inotropes at admission	349 (59.9)	109 (42.4)	240 (73.6)	< 0.0001	
Organ dysfunction scores					
SOFA (points)	7 [5–10]	5 [3–8]	8 [6–11]	< 0.0001	*n* = 509
APACHE II (points)	15 [11–20]	14 [9–17]	18 [13–22]	< 0.0001	
Length of stay (days)					
ICU	5 [2–10]	4 [2–8]	5 [2–10]	0.0554	
Mortality					
28-day, deaths (%)	127 (21.8)	30 (11.7)	97 (29.8)	< 0.0001	
90-day, deaths (%)	166 (28.5)	41 (16)	125 (38.3)	< 0.0001	

*Abbreviations: APACHE* Acute Physiology and Chronic Health Evaluation, *bio-ADM* Bioactive adrenomedullin, *BNP* Brain-derived natriuretic peptide, *BUN* Blood urea nitrogen, *CNS* Central nervous system, *COPD* Chronic obstructive pulmonary disease, *ICU* Intensive care unit, *NT-proBNP* N-terminal brain natriuretic peptide, *PaO*_*2*_*/FiO*_*2*_ Ratio of partial pressure of arterial oxygen to fraction of inspired oxygen, *PCT* Procalcitonin, *SOFA* Sequential Organ Failure Assessment

* *p* Value from nonparametric Kruskal-Wallis or chi-square test, respectively

^a^ Most common comorbidities reported individually

### Bio-ADM levels and mortality

Over the 28-day follow-up period, 127 patients (22%) died: 33 with sepsis and 94 with septic shock.

In a Cox proportional hazards model adjusted for age, gender, comorbidities (cardiac and noncardiac), lactate, and diagnosis (sepsis, septic shock), bio-ADM concentration at admission was independently associated with 28-day mortality in the studied population (added chi-square 12.2, *p* = 0.0005; adjusted standardized HR 1.6 [95% CI 1.1–2.5], *p* = 0.0004) (Table 2). Noticeably, the C index for prediction of 28-day mortality for bio-ADM at admission was 0.688 (95% CI 0.642–0.733, chi-square 54.8, *p* < 0.0001) in the univariate Cox regression. C indexes for lactate, SOFA, and APACHE II were 0.720 (95% CI 0.672–0.768), 0.728 (95% CI 0.680–0.777), and 0.701 (95% CI 0.657–0.746), respectively (all *p* < 0.0001). A multivariate model further demonstrated that bio-ADM had added value on top of APACHE II or SOFA score (added chi-square 24.4 [*p* < 0.0001] and 10.2 [*p* = 0.0014], respectively) (Table 2) when used as a continuous variable.

**Table 2:** Association between bio-ADM and 28-day mortality

Variables	Chi-square	added chi-square	*p* Value (added value)	Std. HR bio-ADM	*p* Value
bio-ADM (univariate)	54.8			2.3 [1.9–2.9]	< 0.0001
Adjusted for SOFA at admission	85.1	10.2	0.0014	1.6 [1.2–2.1]	0.0014
Adjusted for APACHE II at admission	88.9	24.4	< 0.0001	1.9 [1.5–2.4]	< 0.0001
Adjusted for covariates	132.1	12.2	0.0005	1.6 [1.1–2.5]	0.0004
bio-ADM (time-dependent Cox)	80.6	25.8	< 0.0001	2.5 [2.1–3.1]	< 0.0001
Adjusted for SOFA at admission	89.3	11.5	0.0007	1.8 [1.4–2.2]	< 0.0001
Adjusted for APACHE II at admission	108.4	19.5	< 0.0001	2.1 [1.7–2.6]	< 0.0001
Adjusted for SOFA (t-d*)	101.0	7.9	0.0049	1.5 [1.1–2.0]	0.0048
Adjusted for lactate (t-d*)	138.0	35.7	< 0.0001	1.9 [1.5–2.3]	< 0.0001

*APACHE* Acute Physiology and Chronic Health Evaluation II, *bio-ADM* Bioactive adrenomedullin, *SOFA* Sequential Organ Failure Assessment

Results are from uni- (chi-square), multi- (added chi-square), and *time-dependent Cox regression analysis. *Time-dependent analysis includes measurements observed at baseline and day 2. *n* = 562 for covariates (i.e., age, gender, comorbidities [cardiac and noncardiac], diagnosis [sepsis, septic shock], lactate) model due to missing data for time-dependent lactate, and *n* = 509 for models including *time-dependent SOFA score

**Fig. 1:**
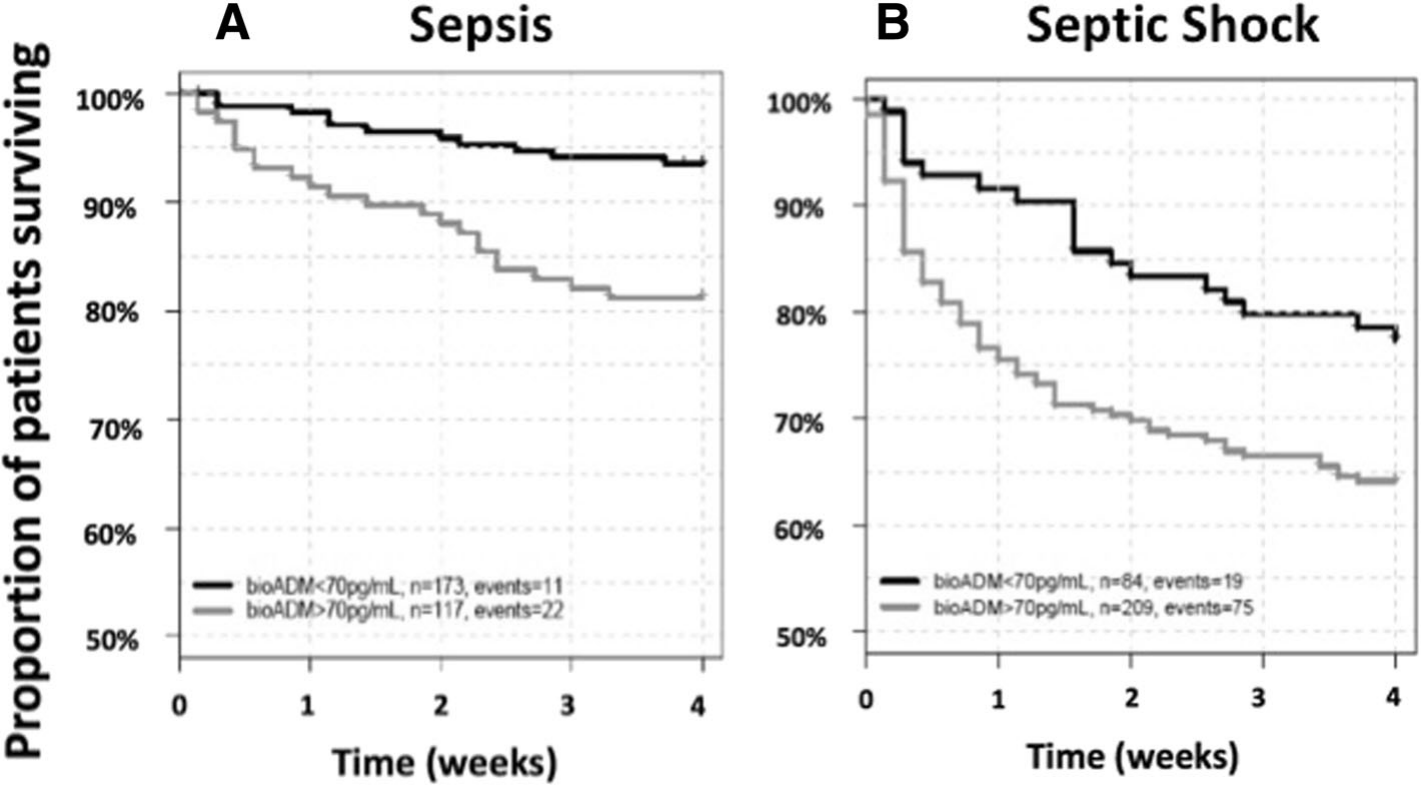
Twenty-eight-day Kaplan-Meier survival curves of low versus high biologically active adrenomedullin at admission, based on a cutoff value of 70 pg/ml, in (**a**) sepsis, and (**b**) septic shock patients

With the predefined cutoff value of 70 pg/ml, Kaplan-Meier analysis confirmed predictive value of bio-ADM for 28-day mortality in all studied patients (Additional file 1: Figure S1) and in subgroups of sepsis and septic shock (Fig. 1a and b). Patient characteristics for high and low bio-ADM levels are illustrated in Table 1, and characteristics for survivors versus nonsurvivors are provided in Additional file 2: Table S1. The optimal Youden cutoff in all patients was 101.9 pg/ml (sensitivity 67.7%, specificity 67.3%). In septic shock, the optimal Youden cutoff was 99.1 pg/ml (sensitivity 71.3%, specificity 52.3%), and in severe sepsis it was 101.9 pg/ml (sensitivity 57.6%, specificity 78.6%). This compares with a sensitivity of 77.2% and specificity of 48.9% in all patients for the predefined bio-ADM cutoff of 70 pg/ml.

**Fig. 2:**
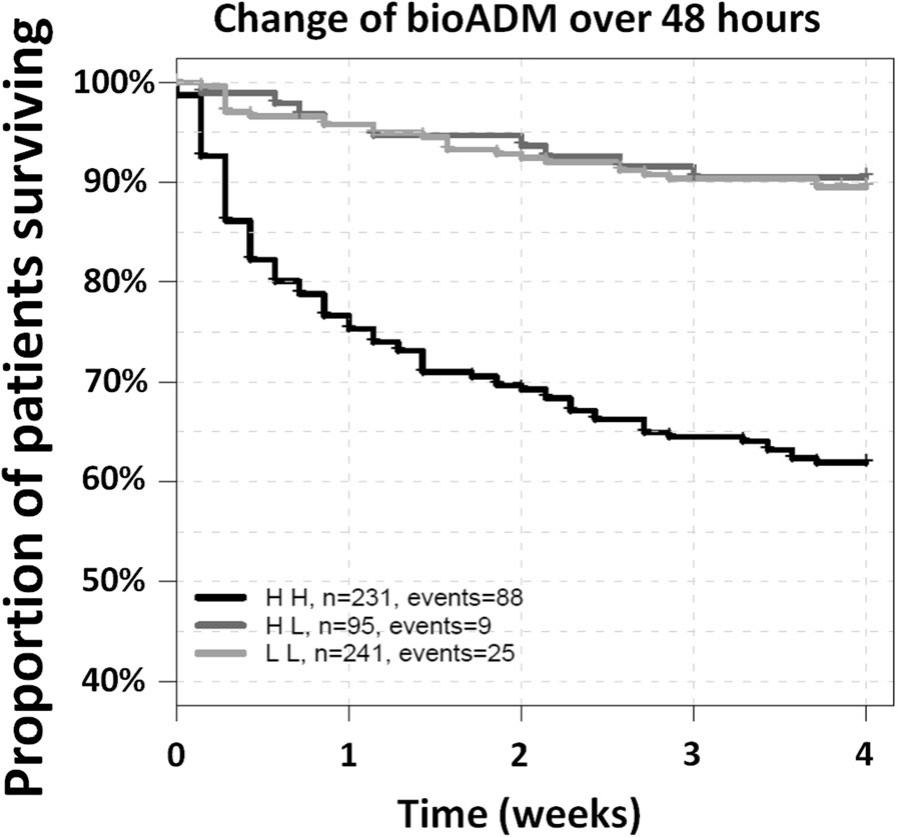
Association between the changes of biologically active adrenomedullin (bio-ADM) levels over 48 h and mortality. HR between high-high (HH) (levels of bio-ADM remained high) and high-low (HL) (levels of bio-ADM declining over 48 h) 4.9 (95% CI 2.5–9.8; HR of LL 1.1 [0.52–2.4]). Only a small number (*n* = 16, 2.7%; 28-day survival rate 68.8%) of patients who presented with a low bio-ADM concentration upon admission had higher bio-ADM level on day 2 (low-high (LH) group), which is why this group is not represented in the figure

We additionally assessed outcome in relation to bio-ADM changes in the initial 48 h in time-dependent Cox regression. Bio-ADM trajectory over the initial 48 h after study inclusion improved prediction of 28-day survival in the overall population (added chi-square 25.8, *p* < 0.0001) (Table 2; Fig. 2**,** Additional file 3: Figure S2) and was independent of time-dependent lactate or SOFA score evaluation (Table 2). Patients were divided into four groups based on baseline and day 2 bio-ADM concentrations and under implementation of the cutoff value of 70 pg/ml: remaining low (low-low, LL), high-to-low (HL), low-to-high (LH), and remaining high (high-high, HH). Patient characteristics of these subgroups are presented in Additional file 4: Table S2.

In patients admitted with high bio-ADM upon admission, those who decreased bio-ADM towards normal values within the first 48 h (HL group) had a similar 28-day mortality to the LL group (HL 9.5%, LL 10.5%) and a more favorable outcome than patients whose bio-ADM remained high (HH group) or became high (LH group) (28-day mortality of 38.1% and 38.2%) (Additional file 4: Table S2).

### Bio-ADM levels and organ dysfunction

**Fig. 3:**
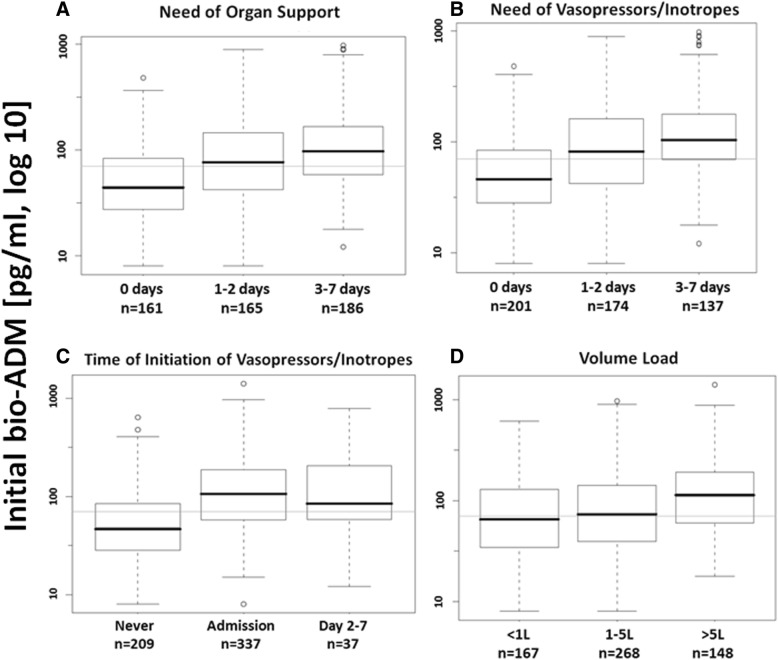
Association between biologically active adrenomedullin levels upon admission and (**a**) length of total organ support over the first 7 days (*p* < 0.0001), (**b**) length of vasopressor/inotropic support over the first 7 days (*p* < 0.0001), (**c**) overall need for vasopressor support (*p* < 0.0001), and (**d**) total fluid balance over the initial 48 h (*p* = 0.0001)

Bio-ADM levels upon admission correlated with the initial SOFA score in all studied patients (*n* = 509, *r* = 0.49, *p* < 0.0001) (Additional file 5: Figure S3). SOFA score was higher in patients in septic shock than in those in sepsis, and for each group in patients with high initial bio-ADM (Additional file 6: Figure S4). Figure 3a indicates that the initial level of circulating bio-ADM relates to the need for and duration of organ support in survivors (*p* < 0.0001).

Concerning circulating bio-ADM levels and cardiovascular function, we found an almost linear relationship of bio-ADM and both cardiovascular SOFA subscore (*p* < 0.001) (Additional file 7: Figure S5) and duration of cardiovascular drug support (Fig. 3b) (*p* < 0.0001). Understandably, patients with high bio-ADM needed norepinephrine at admission more frequently (73% versus 42%, *p* < 0.0001) and at greater dose (0.4 [0.3–0.8] versus 0.2 [0.1–0.4] μg/kg/min, *p* = 0.0022) than patients with low bio-ADM (Additional file 8: Table S3). Our analysis further revealed that patients with high bio-ADM at admission needed more vasopressors/inotropes over the following 7 days even if they did not have those treatments at admission (Fig. 3c).

Regarding other organ support, patients who needed volume resuscitation of more than 5 L over the first 2 days (Fig. 3d) (*p* < 0.0001) or RRT (Additional file 9: Figure S6) or had long ICU stay (Additional file 10: Figure S7) had much higher circulating bio-ADM levels upon ICU admission than those patients who did not.

**Fig. 4:**
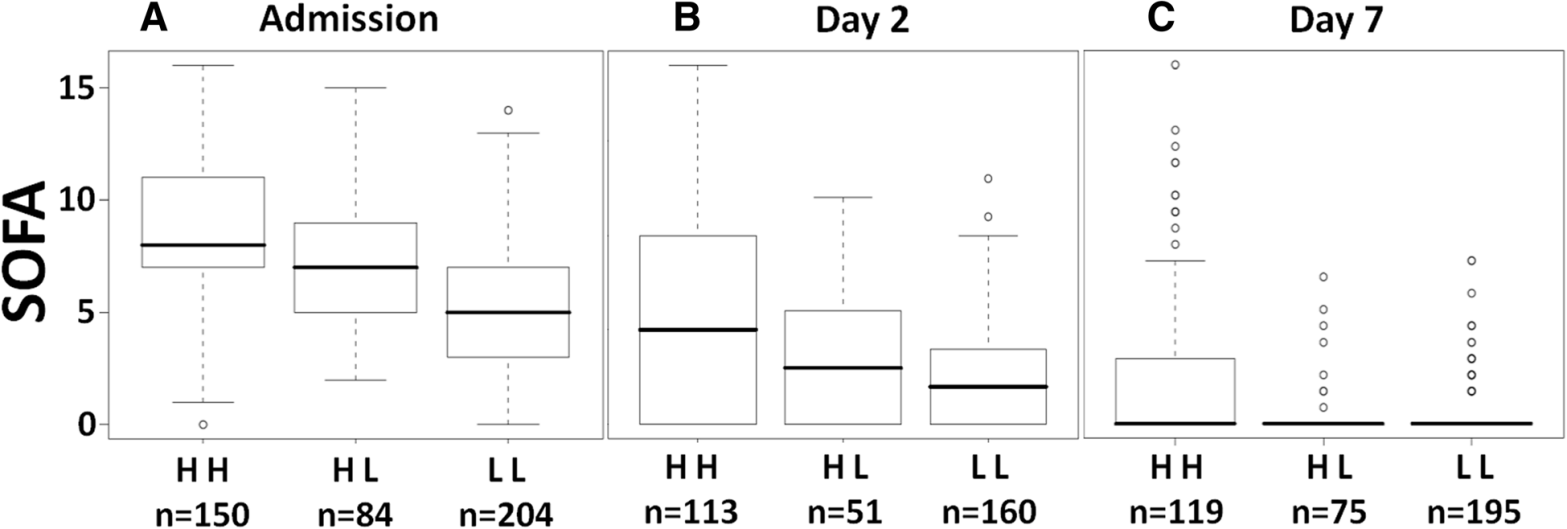
The absolute Sequential Organ Failure Assessment (SOFA) scores at (**a**) admission, (**b**) day 2, and (**c**) day 7 for groups high-high (HH; i.e., above 70 pg/ml at baseline and day 2), high-low (HL), and low-low (LL), excluding patients who died within 7 days. *p* Value for differences between HH vs. LL: *p* < 0.0001 for all days; *p* value for HH vs. HL: *p* < 0.0001 for all days; *p* Values for HL vs. LL: *p* < 0.0001, 0.6016, and 0.9969 for days 1, 3, and 7, respectively. Of note, the number of patients is less at day 2 than at day 7 because there were more values missing at day 2 owing to the fact that discharged patients (mostly at day 7) were given a SOFA score of 0. Furthermore, only a small number (*n* = 16, 2.7%) of patients who presented with a low bio-ADM concentration upon admission had a higher bio-ADM level on day 2 (low-high [LH] group), which is why this group is not represented in the figure. Median (IQR) SOFA scores for the LH group were 7.5 (6.0–9.8), 9.0 (4.0–11.2), and 4.0 (0.0–6.5) for admission, day 2, and day 7, respectively

In agreement with the fact that serial measurements of bio-ADM indicated survival benefit in patients who dropped bio-ADM levels at day 2, we could demonstrate that drop of bio-ADM over the first 2 days also preceded the decrease of total SOFA score (*p* value for differences between HH vs. HL: *p* < 0.0001 for all days) (Fig. 4).

Finally, using the Sepsis-3 definition of septic shock (i.e., vasopressor use and lactate ≥ 2mmol/L [or 18 mg/dl] despite adequate volume resuscitation [17]), our analysis confirmed that bio-ADM upholds a strong prognostication for organ recovery and survival in AdrenOSS-1 (both *p* < 0.0001) (Additional file 11: Figure S8A and B).

## Discussion

The AdrenOSS-1 study was a prospective multinational observational cohort study assessing the relationship between rapid changes in circulating bio-ADM levels in the first 2 days and clinical outcome in ICU patients with sepsis and septic shock. We confirmed elevated levels of bio-ADM in septic patients and the striking relationship between circulating bio-ADM at ICU admission, organ dysfunction, and death. We also demonstrated that early recovery of circulating bio-ADM levels towards normal values (i.e., < 70 pg/ml) was associated with normalization of vascular function and better 28-day survival.

Our study found moderately elevated circulating levels of bio-ADM at admission in sepsis and strongly elevated bio-ADM levels in patients with septic shock, in accordance with earlier reports [13, 14]. Our study also confirmed the marked association between bio-ADM level at admission and short-term mortality as well as the prognostic cutoff value of 70 pg/ml, previously described by Marino et al. [14] and Caironi et al. [13] in both sepsis and septic shock (including the most recent definition [17]). Our study showed moderate prognostic value of bio-ADM at admission using AUC but marked prognostic value using Cox proportional hazards model adjusted for various parameters. Moreover, our study showed that prognostic value of bio-ADM at ICU admission exerts additive value (positive changes in chi-square) to various ICU severity scores. We described also the association between a bio-ADM ≤ 70 pg/ml on day 2 and very low 28-day mortality, even in patients with initial high bio-ADM levels. The association of low bio-ADM by day 2 with full restoration of organ function at day 7 has been shown as well.

Concerning organ dysfunction, we found a relationship between circulating bio-ADM at ICU admission and the subsequent need for cardiovascular and/or renal support. In our studied patients, high circulating bio-ADM—known to have vasodilatory actions—might account for the deterioration of vascular tone and blood pressure, as previously described [13, 14]. In the present study, patients with high bio-ADM levels on ICU admission were more likely to need vasopressors and/or inotropes either at admission or in the following days. Moreover, they had a higher total fluid balance and higher incidence of RRT during their ICU stay. The ADM-induced vascular dysfunction may have contributed to this condition, although some data suggest that high bio-AM levels might also be protective to the kidney [19, 20]. Further studies are needed to elucidate the exact role of bio-ADM in renal function. Of interest, the relationship between circulating bio-ADM levels and extent of organ dysfunction, present during ICU admission, was also true during the recovery phase. Indeed, bio-ADM levels decreased before the improvement of total SOFA score in our investigation. Patients with high bio-ADM levels at ICU admission who showed a decline towards normal bio-ADM values at day 2 were more likely to recover vascular function and vasopressor need by day 7. By contrast, the drop in bio-ADM from ICU admission to day 2 was associated with only limited improvement in renal function or no improvement in lung function at day 7. These observations also warrant further exploration.

Circulating bio-ADM levels were lower in AdrenOSS-1 than in the previously described ALBIOS cohort [13]. Indeed, in ALBIOS, septic patients were more severe, as suggested by greater prevalence of mechanical ventilation, length of stay, and short-term mortality. Likewise, the prevalence of septic shock was greater in ALBIOS than in AdrenOSS-1 (Additional file 12: Table S4). Of note, different definitions of septic shock in the two studies may have influenced study assessments.

Limitations included that in the present population only patients with sepsis and septic shock were studied, and results cannot be directly translated to a general ICU population. Future studies should focus on extrapolation of our results to patients with hemodynamic instability related to other disease, because as study has already been performed for cardiogenic shock [21]. Furthermore, our data suggest that ADM may be associated with myocardial function (e.g., patients with high ADM also had significantly higher circulating natriuretic peptide levels). However, data on cardiac function (e.g., cardiac output or left ventricular ejection fraction) were available in only few studied patients. Finally, we used the cut point of 70 pg/ml of circulating Bio-ADM for validation of the previously published cut point, even though the optimal Youden cut points in AdrenOSS-1 showed that 70 pg/ml with respect to a technical optimality criterion is not optimal.

Strong points of the study are the fact that it was a prospective international multicenter study with a large number of patients, with a focus on mortality and organ dysfunction. However, as is true of any observational study, only associations can be described, and cause-and-effect relationships cannot be deducted.

## Conclusions

In this large prospective international cohort of critically ill patients admitted to the ICU with sepsis or septic shock, we confirmed the strict relationship between high levels of bio-ADM at ICU admission and organ dysfunction and mortality. We demonstrated that early decrease towards the normal values of circulating bio-ADM in the first days after ICU admission was associated with improvement of cardiovascular and renal function and was associated with very low 28-day mortality.

### Additional files

Additional file 1: **Figure S1.** Twenty-eight-day Kaplan-Meier survival curves of low versus high bio-ADM at admission (bioADM.d0) in all patients, based on a cutoff value of 70 pg/ml. (TIF 207 kb)

Additional file 2: **Table S1.** Patient characteristics of survivors and nonsurvivors. (DOCX 50 kb)

Additional file 3: **Figure S2.** Bio-ADM levels at baseline and on day 2 in 28-day survivors and nonsurvivors. If data were missing at day 2 (e.g., owing to death or discharge; 12.7%), the last available measurement was carried forward. Horizontal lines at 70 and 130 pg/ml for better orientation; *y*-axis is truncated at 300 pg/ml. (TIF 206 kb)

Additional file 4: **Table S2.** Patient characteristics of the four different groups with respect to adrenomedullin trajectory over the first 48 h after study inclusion. (DOCX 56 kb)

Additional file 5: **Figure S3.** Association between the initial bio-ADM concentration and initial SOFA score (*r* = 0.49, *n* = 509, *p* < 0.0001; missing values due to missing SOFA score components). (TIF 199 kb)

Additional file 6: **Figure S4.** Association of initial SOFA score by sepsis and septic shock and initial bio-ADM concentration below or above 70 pg/ml (*p* < 0.0001 for both bio-ADM and diagnosis; *p* = 0.2015 for interaction; two-way analysis of variance). All data are from admission. (TIF 195 kb)

Additional file 7: **Figure S5.** Relationship between bio-ADM and cardiovascular SOFA subscore (*p* < 0.001). (JPG 25 kb)

Additional file 8: **Table S3.** Association between adrenomedullin and need of vasopressors/inotropes at admission. (DOCX 23 kb)

Additional file 9: **Figure S6.** Association between bio-ADM concentration on admission and need for renal replacement therapy on admission, later during ICU stay, or never (70.4 [36.3–128.8] vs. 149.0 [87.1–320.5] and 162.6 [99.8–367.3] pg/ml, for patients without need for RRT, on admission, or later during ICU stay, respectively, *p* < 0.0001). (TIF 221 kb)

Additional file 10: **Figure S7.** Bio-ADM levels upon admission in 28-day survivors and time to ICU discharge (*p* < 0.0001): Patients with early discharge (< 2 days) are significantly different from all other groups (all *p* < 0.016), and late discharge (> 21 days) is significantly different from early discharge (< 2 days and 2–7 days, both *p* < 0.013). (TIF 217 kb)

Additional file 11: **Figure S8.** Twenty-eight-day Kaplan-Meier survival curves of low versus high bio-ADM at admission, based on a cutoff value of 70 pg/ml, in patients with lactate > 2 mmol/L (*p* < 0.0001) (**a**) and SOFA score (**b**) for low versus high bio-ADM at admission (*p* < 0.0001). (TIF 229 kb)

Additional file 12: **Table S4.** Comparison of AdrenOSS-1 and ALBIOS. (DOCX 24 kb)

## Appendix

### Collaborators

**Belgium,** Brussels: Pierre-François Laterre, Caroline Berghe, Marie-France Dujardin, Suzanne Renard, Xavier Wittebole, Christine Collienne, Diego Castanares Zapatero; Ottignies: Thierry Dugernier, Marco Vinetti, Nicolas De Schryver, Anne Thirifays, Jacques Mairesse; Haine-St-Paul: Vincent Huberlant, Hélène Petre, Isabelle Buelens, Pierre Henin, Hugues Trine, Yves Laurent, Loix Sébastien, Paul Geukens, Laurent Kehl. **France,** Limoges: Bruno François, Philippe Vignon, Nicolas Pichon, Emmanuelle Begot, Anne-Laure Fedou, Catherine Chapellas, Antoine Galy, Nicolas Rodier, Ludmilla Baudrillart, Michelle Nouaille, Séverine Laleu, Claire Mancia, Thomas Daix, Paul Bourzeix, Isabelle Herafa, Anne-Aurore Duchambon; La Roche sur Yon: Jean Baptiste Lascarrou, Maud Fiancette, Gwenhael Colin, Matthieu Henry-Lagarrigue, Jean-Claude Lacherade, Christine Lebert, Laurent Martin-Levèvre, Isabelle Vinatier, Aihem Yehia, Konstantinos Bachoumas, Aurélie Joret, Jean Reignier, Cécille Rousseau, Natacha Maquigneau, Yolaine Alcourt, Vanessa Erragne Zinzonni, Angélique Deschamps, Angelina Robert; Tours: Emmanuelle Mercier, Véronique Simeon-Vieules, Aurélie Aubrey, Christine Mabilat, Denis Garot, Stephan Ehrmann, Annick Legras, Manikikian, Youenn Jouan, Pierre-François Dequin, Antoine Guillon, Laetitia Bodet-Contentin, Emmannuelle Rouve, Charlotte Salmon, Lysiane Brick, Stéphanie Massat; Angoulême: Arnaud Desachy, Marie Anne Fally, Laurence Robin, Christophe Cracco, Charles Lafon, Sylvie Calvat, Stéphane Rouleau, David Schnell; Angers: Sigismond Lasocki, Philippe Fesard, Damien Leblanc, Guillaume Bouhours, Claire Chassier, Mathieu Conte, Thomas Gaillard, Floriane Denou, Mathieu Kerymel, Marion Guyon, Anthéa Loiez, Stéphanie Lebreton; Strasbourg – Nouvel Hôpital Civil: Ferhat Meziani, Hayat Allam, Samir Chenaf, Hassène Rahmani, Sarah Heenen, Christine Kummerlen, Xavier Delabranche, Alexandra Boivin, Raphaël Clere-Jehl, Yannick Rabouël; Strasbourg – Hôpital HautePierre: Julien Pottecher, Sophie Bayer, Catherine Metzger, Stéphane Hecketsweiler, Pierre Olivier Ludes, Hortense Besancenot, Nadia Dhif, Guy Freys, Jean-Marc Lessinger, Anne Launoy, Aude Ruimy, Alain Meyer, M Szozot; Paris – Hôpital Lariboisière: Alexandre Mebazaa, Nicolas Deye, Etienne Gayat, Marie-Céline Fournier, Sarra Abroug, Badr Louadah, Elodie Feliot, Sebastian Voicu, Malissin I, Bruno Megarbane, Philippe Manivet, Gardianot Victori, DaSilva Kelly, Béatrice Foucher, Valérie Pierre, Lamia Kerdjana, Thomas Beeken, Antoine Goury, Pierre Garcon, Samuel Gaugain, Benjamin Glen Chousterman, Benjamin Huot, Romain Barthelemy, Benjamin Soyer; Paris – Hôpital St Louis: Laurent Jacob, Matthieu Legrand, Marie-Céline Fournier, Francine Bonnet, Chloé Legall, Haikel Oueslati, Alexandru Cupaciu, Philippe Manivet, Badr Louadah; Paris – Hôpital Bichat: Romain Sonneville, Sophie Letrou, Lila Bouadma, Bruno Mourvillier, Véronique Deiler, Eric Magalhaes, Mathilde Neuville, Jean-François Timsit, Aguila Radjou; Colombes: Stéphane Gaudry, Emeline Dubief, Jonathan Messika, Béatrice La Combe, Damien Roux, Guillaume Berquier, Mohamed Laissi, Jean-Damien Ricard; Clermont Ferrand: Jean-Michel Constantin, Sebastien Perbet, Julie Delmas, Julien Pascal, Sophie Cayot, Renaud Guerin, Matthieu Jabaudon, Laurence Roszyk, Christine Rolhion, Justine Bourdier, Mathilde Lematte, Charlène Gouhier, Camille Verlhac, Thomas Godet, Sophiano Radji, Elodie Caumon, Sandrine Thibault. **Germany,** Aachen: Nikolaus Marx, Tobias Schuerholz, Jessica Pezechk, Florian Feld, Christian Brülls, Thorben Beeker, Tim-Philipp Simon, Robert Deisz, Achim Schindler, Bianca Meier, Thorsten Janisch; Köln: Andreas Hohn, Dirk Schedler, Wolfgang Wetsch, Daniel Schröder; Erfurt: Andreas Meier-Hellmann, Alexander Lucht, Robert Henker, Magdalena Römmer, Torsten Meinig; Frankfurt: Kai D. Zacharowski, Patrick Meybohm, Simone Lindau, Haitham Mutlak; Hamburg: Stefan Kluge, Grit Ringeis, Birgit Füllekrug, Brigitte Singer, Axel Nierhaus, Katrin Bangert, Geraldine de Heer, Daniel Frings, Valentin Fuhrmann, Jakob Müller, Jörg Schreiber, Barbara Sensen, Stephanie Siedler, Annekatrin Siewecke, Gerold Söffker, Dominic Wichmann, Mélanie Kerinn; Augsburg: Ulrich Jaschinski, Ilse Kreuser, Marlene Zanquila; Jena: Andreas Kortgen, Frank Bloos, Falk Gonnert, Daniel Thomas-Rüddel, Anja Haucke, Steffi Kolanos, Karina Knuhr Kohlberg, Petra Bloos, Katrin Schwope. **Italy,** Rome: Sant’Andrea Hospital: Salvatore Di Somma, Marino Rossella, Veronica Russo, Santarelli Simona, Christopher Bartoli, Sylvia Navarin, Cristina Bongiovanni, Michela Orru, Daniela Quatrocchi, Giada Zoccoli, Antonella Varchetta; Rome – Policlinico Universitario A. Gemelli: Massimo Antonelli, Gennaro de Pascale, Maria Sole Vallecoccia, Salvatore Lucio Cutuli, Valentina Digravio, Daniela Quattrochi, Sonia D’Arrigo, Filippo Elvino Leone. **The Netherlands,** Enschede: Bert Beishuizen, Martin Rinket, Natalie Border, Mariska Bos-Burgmeijer, Astrid Braad, S Papendorp, Alexander Cornet, J Vermeijden, Ronald J Trof; Nijmegen: Peter Pickkers, Marieke van de A, Helen Van Wezel, Leo Heunks, Natalie Border, Chantal Luijten-Arts, Astrid Hoedemaekers, Hans van der Hoeven, Noortje Roovers, Pleun Hemelaar.

**Annex: Sponsoring**

**sphingotec GmbH**

Neuendorfstraße 15a

16761 Hennigsdorf

Germany

**Annex: Management**

**European Drug Development Hub (EDDH),** Vandoeuvres Les Nancy: Stéphanie Grojean, Laetitia Tourneur, Virginie Barthel

## Abbreviations

ADM: Adrenomedullin
AdrenOSS: Adrenomedullin and Outcome in Sepsis and Septic Shock
APACHE II: Acute Physiologic Assessment and Chronic Health Evaluation II
bio-ADM: Biologically active adrenomedullin
BNP: Brain-derived natriuretic peptide
BUN: Blood urea nitrogen
CNS: Central nervous system
COPD: Chronic obstructive pulmonary disease
ICU: Intensive care unit
NT-proBNP: N-terminal brain natriuretic peptide
PaO_2_/FiO_2_: Ratio of partial pressure of arterial oxygen to fraction of inspired oxygen
PCT: Procalcitonin
RRT: Renal replacement therapy
SOFA: Sequential Organ Failure Assessment

## References

[1] Kox M, Pickkers P (2014). Adrenomedullin: its double-edged sword during sepsis slices yet again. Intensive Care Med Exp.

[2] Nuki C, Kawasaki H, Kitamura K, Takenaga M, Kangawa K, Eto T, Wada A (1993). Vasodilator effect of adrenomedullin and calcitonin gene-related peptide receptors in rat mesenteric vascular beds. Biochem Biophys Res Commun.

[3] Passaglia P, Gonzaga NA, Tirapelli DP, Tirapelli LF, Tirapelli CR (2014). Pharmacological characterisation of the mechanisms underlying the relaxant effect of adrenomedullin in the rat carotid artery. J Pharm Pharmacol.

[4] Nakamura M, Yoshida H, Makita S, Arakawa N, Niinuma H, Hiramori K (1997). Potent and long-lasting vasodilatory effects of adrenomedullin in humans: comparisons between normal subjects and patients with chronic heart failure. Circulation.

[5] Hippenstiel S, Witzenrath M, Schmeck B, Hocke A, Krisp M, Krüll M, Seybold J, Seeger W, Rascher W, Schütte H (2002). Adrenomedullin reduces endothelial hyperpermeability. Circ Res.

[6] Brell B, Temmesfeld-Wollbruck B, Altzschner I, Frisch E, Schmeck B, Hocke AC, Suttorp N, Hippenstiel S (2005). Adrenomedullin reduces *Staphylococcus aureus* alpha-toxin-induced rat ileum microcirculatory damage. Crit Care Med.

[7] García Ponce A, Citalán Madrid AF, Vargas Robles H, Chánez Paredes S, Nava P, Betanzos A, Zarbock A, Rottner K, Vestweber D, Schnoor M (2016). Loss of cortactin causes endothelial barrier dysfunction via disturbed adrenomedullin secretion and actomyosin contractility. Sci Rep.

[8] Temmesfeld-Wollbruck B, Brell B, David I, Dorenberg M, Adolphs J, Schmeck B, Suttorp N, Hippenstiel S (2007). Adrenomedullin reduces vascular hyperpermeability and improves survival in rat septic shock. Intensive Care Med.

[9] Hocke AC, Temmesfeld-Wollbrueck B, Schmeck B, Berger K, Frisch EM, Witzenrath M, Brell B, Suttorp N, Hippenstiel S (2006). Perturbation of endothelial junction proteins by *Staphylococcus aureus* alpha-toxin: inhibition of endothelial gap formation by adrenomedullin. Histochem Cell Biol.

[10] Muller HC, Witzenrath M, Tschernig T, Gutbier B, Hippenstiel S, Santel A, Suttorp N, Rosseau S (2010). Adrenomedullin attenuates ventilator-induced lung injury in mice. Thorax.

[11] Guignant C, Voirin N, Venet F, Poitevin F, Malcus C, Bohe J, Lepape A, Monneret G (2009). Assessment of pro-vasopressin and pro-adrenomedullin as predictors of 28-day mortality in septic shock patients. Intensive Care Med.

[12] Christ-Crain M, Morgenthaler NG, Struck J, Harbarth S, Bergmann A, Muller B (2005). Mid-regional pro-adrenomedullin as a prognostic marker in sepsis: an observational study. Crit Care.

[13] Caironi P, Latini R, Struck J, Hartmann O, Bergmann A, Maggio G, Cavana M, Tognoni G, Pesenti A, Gattinoni L (2017). Circulating biologically active adrenomedullin (bio-ADM) predicts hemodynamic support requirement and mortality during sepsis. Chest.

[14] Marino R, Struck J, Maisel AS, Magrini L, Bergmann A, Di Somma S (2014). Plasma adrenomedullin is associated with short-term mortality and vasopressor requirement in patients admitted with sepsis. Crit Care.

[15] Geven C, Kox M, Pickkers P (2018). Adrenomedullin and adrenomedullin-targeted therapy as treatment strategies relevant for sepsis. Front Immunol..

[16] Levy MM, Fink MP, Marshall JC, Abraham E, Angus D, Cook D, Cohen J, Opal SM, Vincent JL, Ramsay G (2003). 2001 SCCM/ESICM/ACCP/ATS/SIS International Sepsis Definitions Conference. Crit Care Med.

[17] Singer M, Deutschman CS, Seymour CW, Shankar-Hari M, Annane D, Bauer M, Bellomo R, Bernard GR, Chiche JD, Coopersmith CM (2016). The Third International Consensus Definitions for Sepsis and Septic Shock (Sepsis-3). JAMA.

[18] Weber J, Sachse J, Bergmann S, Sparwaßer A, Struck J, Bergmann A. Sandwich immunoassay for bioactive plasma adrenomedullin. J Appl Lab Med. 2017;2(2):222-33. 10.1373/jalm.2017.023655. 10.1373/jalm.2017.023655 32630976

[19] Inal S, Koc E, Ulusal-Okyay G, Pasaoglu OT, Isik-Gonul I, Oz-Oyar E, Pasaoglu H, Guz G (2014). Protective effect of adrenomedullin on contrast induced nephropathy in rats. Nefrologia.

[20] Oyar EO, Kiris I, Gulmen S, Ceyhan BM, Cure MC, Delibas N, Lortlar N, Okutan H (2012). The protective effect of adrenomedullin on renal injury, in a model of abdominal aorta cross-clamping. Thorac Cardiovasc Surg.

[21] Tolppanen H, Rivas-Lasarte M, Lassus J, Sans-Rosello J, Hartmann O, Lindholm M, Arrigo M, Tarvasmaki T, Kober L, Thiele H (2017). Adrenomedullin: a marker of impaired hemodynamics, organ dysfunction, and poor prognosis in cardiogenic shock. Ann Intensive Care.

