# Supplementary figures and images for: Circulating adrenomedullin estimates survival and reversibility of organ failure in sepsis: the prospective observational multinational Adrenomedullin and Outcome in Sepsis and Septic Shock-1 (AdrenOSS-1) study
Source: Crit Care. 2018 Dec 21;22:354. doi: 10.1186/s13054-018-2243-2 (PMC6305573; doi:10.1186/s13054-018-2243-2)

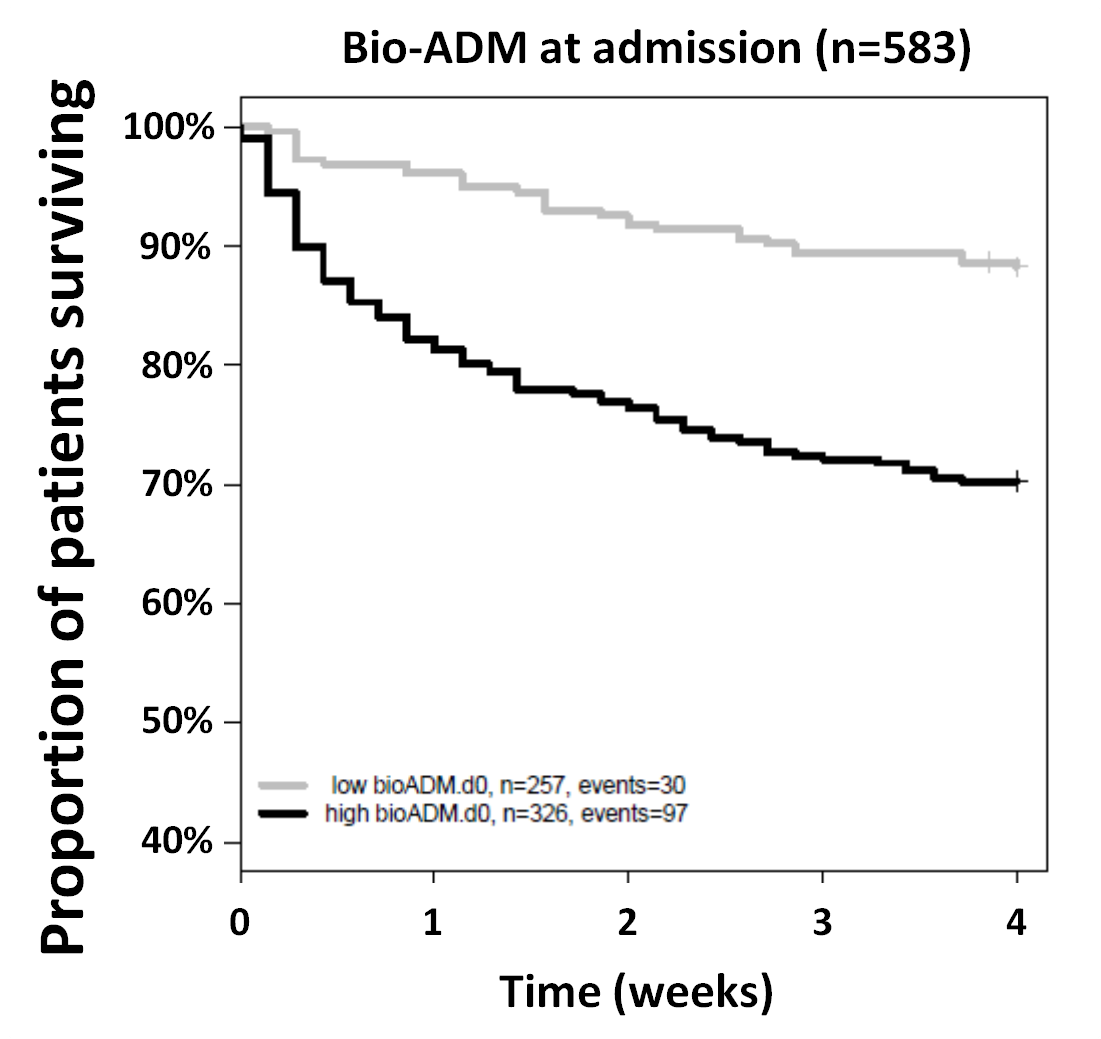

Supplement: Supplementary file 1 — Figure S1. Twenty-eight-day Kaplan-Meier survival curves of low versus high bio-ADM at admission (bioADM.d0) in all patients, based on a cutoff value of 70 pg/ml. (TIF 207 kb) [file 13054_2018_2243_MOESM1_ESM.tif]

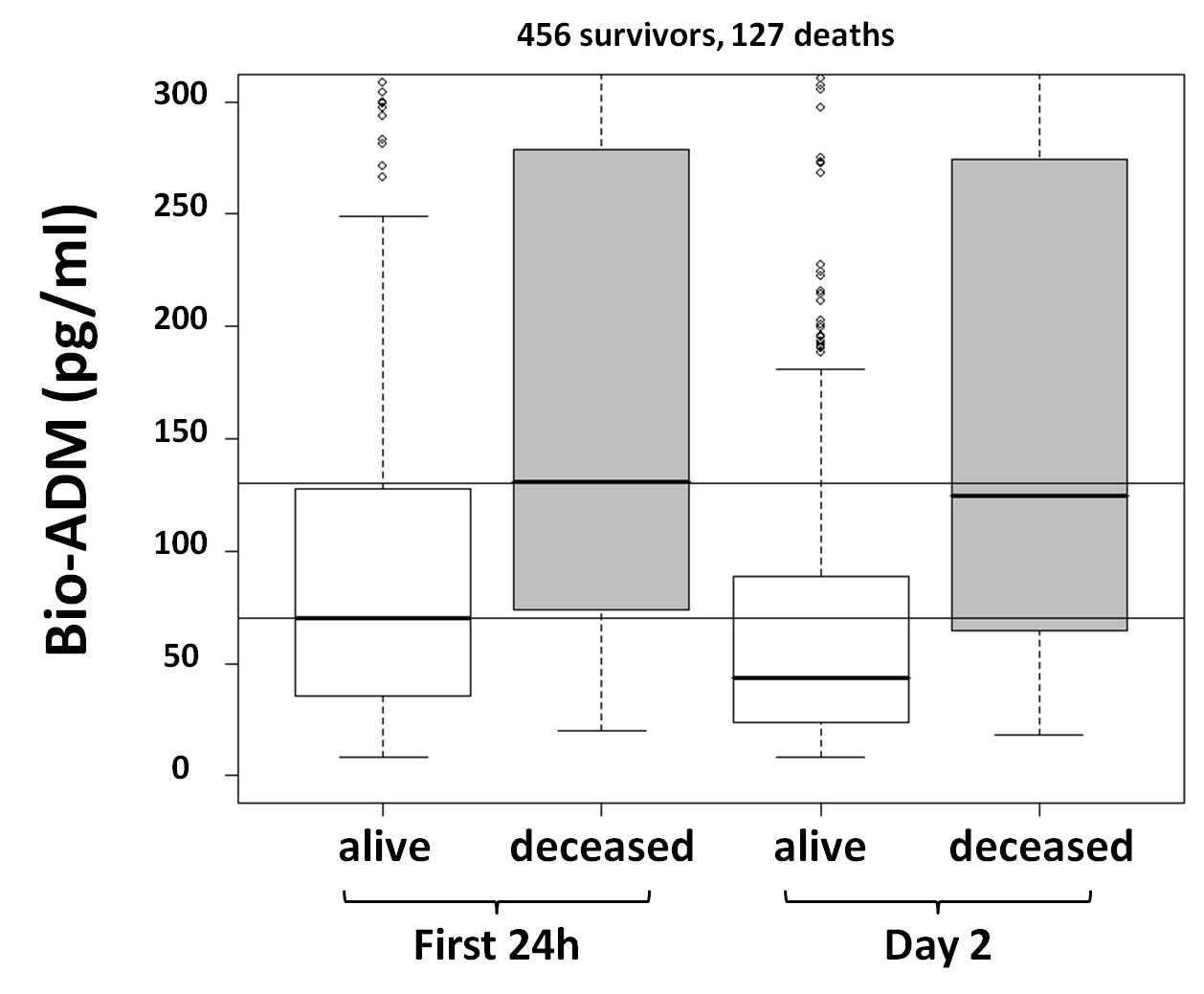

Supplement: Supplementary file 3 — Figure S2. Bio-ADM levels at baseline and on day 2 in 28-day survivors and nonsurvivors. If data were missing at day 2 (e.g., owing to death or discharge; 12.7%), the last available measurement was carried forward. Horizontal lines at 70 and 130 pg/ml for better orientation; y-axis is truncated at 300 pg/ml. (TIF 206 kb) [file 13054_2018_2243_MOESM3_ESM.tif]

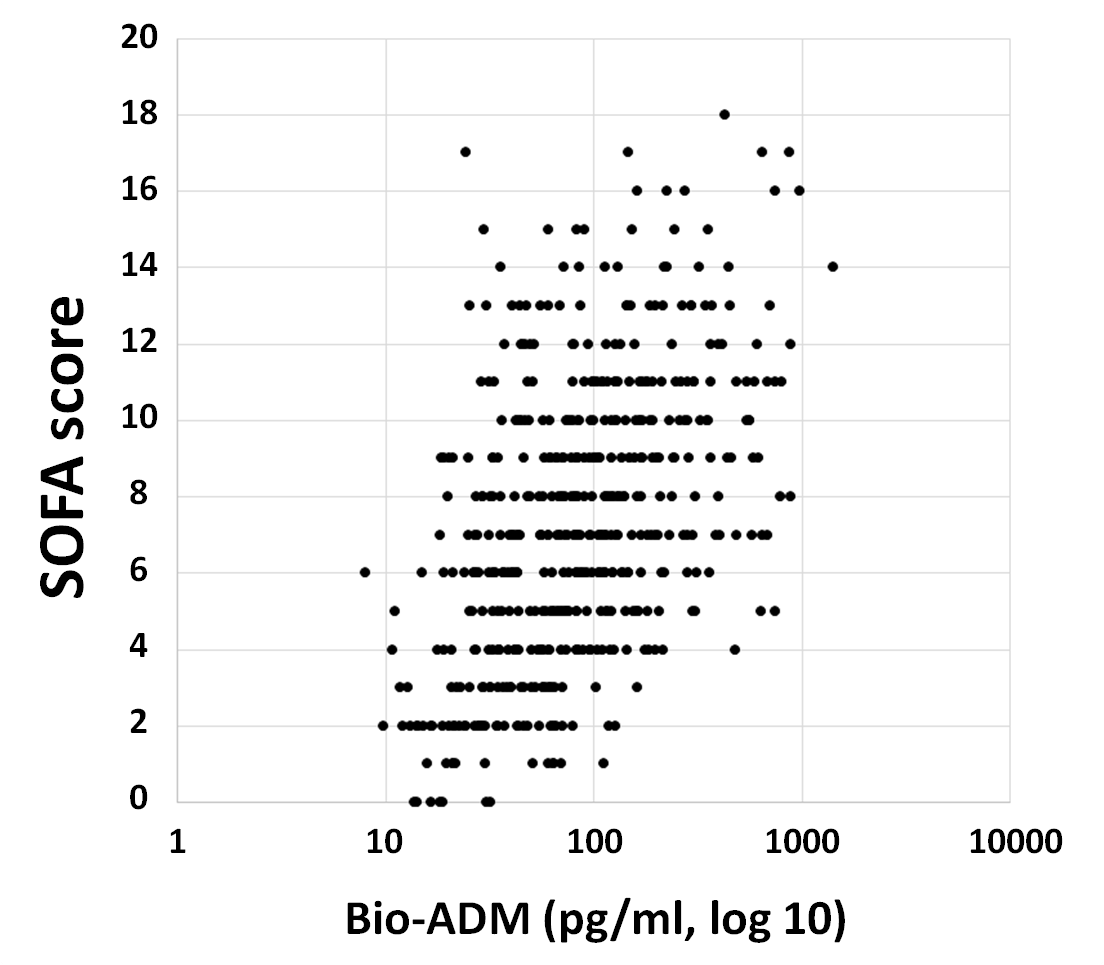

Supplement: Supplementary file 5 — Figure S3. Association between the initial bio-ADM concentration and initial SOFA score (r = 0.49, n = 509, p < 0.0001; missing values due to missing SOFA score components). (TIF 199 kb) [file 13054_2018_2243_MOESM5_ESM.tif]

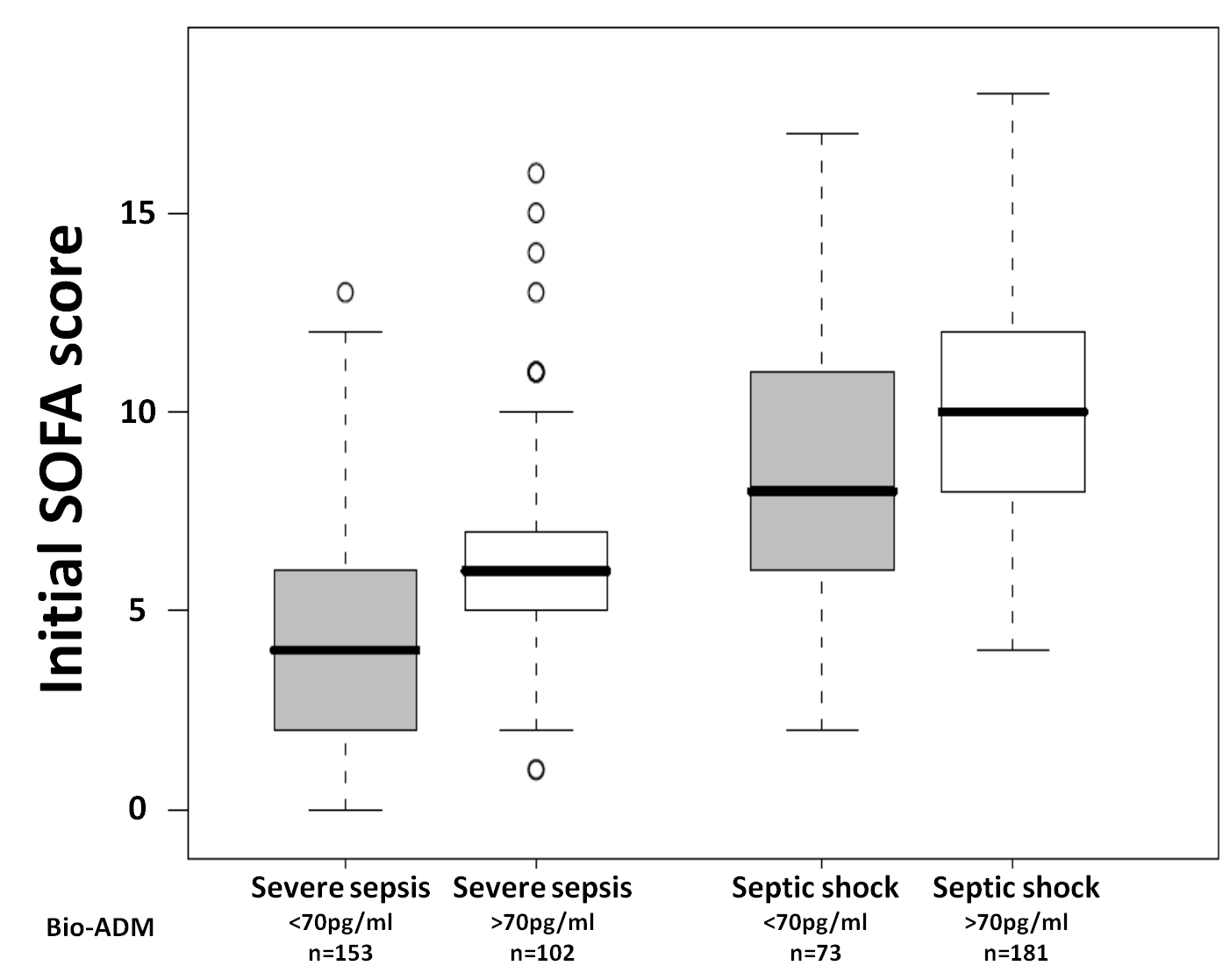

Supplement: Supplementary file 6 — Figure S4. Association of initial SOFA score by sepsis and septic shock and initial bio-ADM concentration below or above 70 pg/ml (p < 0.0001 for both bio-ADM and diagnosis; p = 0.2015 for interaction; two-way analysis of variance). All data are from admission. (TIF 195 kb) [file 13054_2018_2243_MOESM6_ESM.tif]

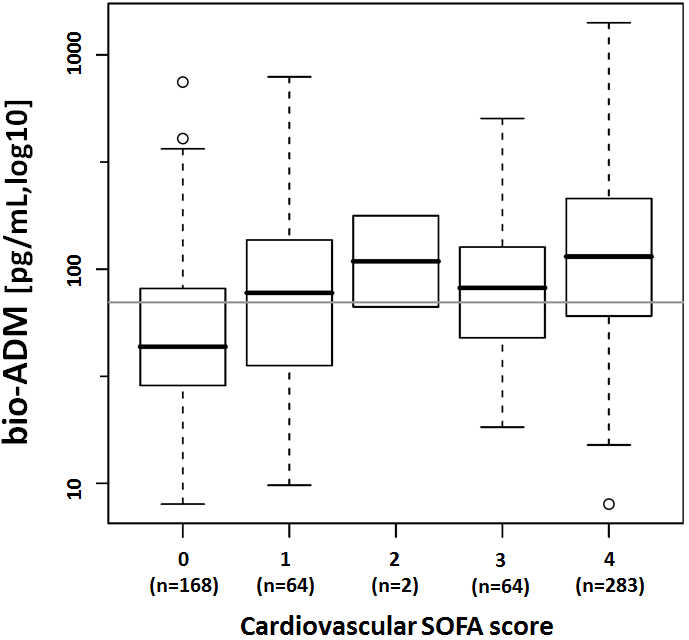

Supplement: Supplementary file 7 — Figure S5. Relationship between bio-ADM and cardiovascular SOFA subscore (p < 0.001). (JPG 25 kb) [file 13054_2018_2243_MOESM7_ESM.jpg]

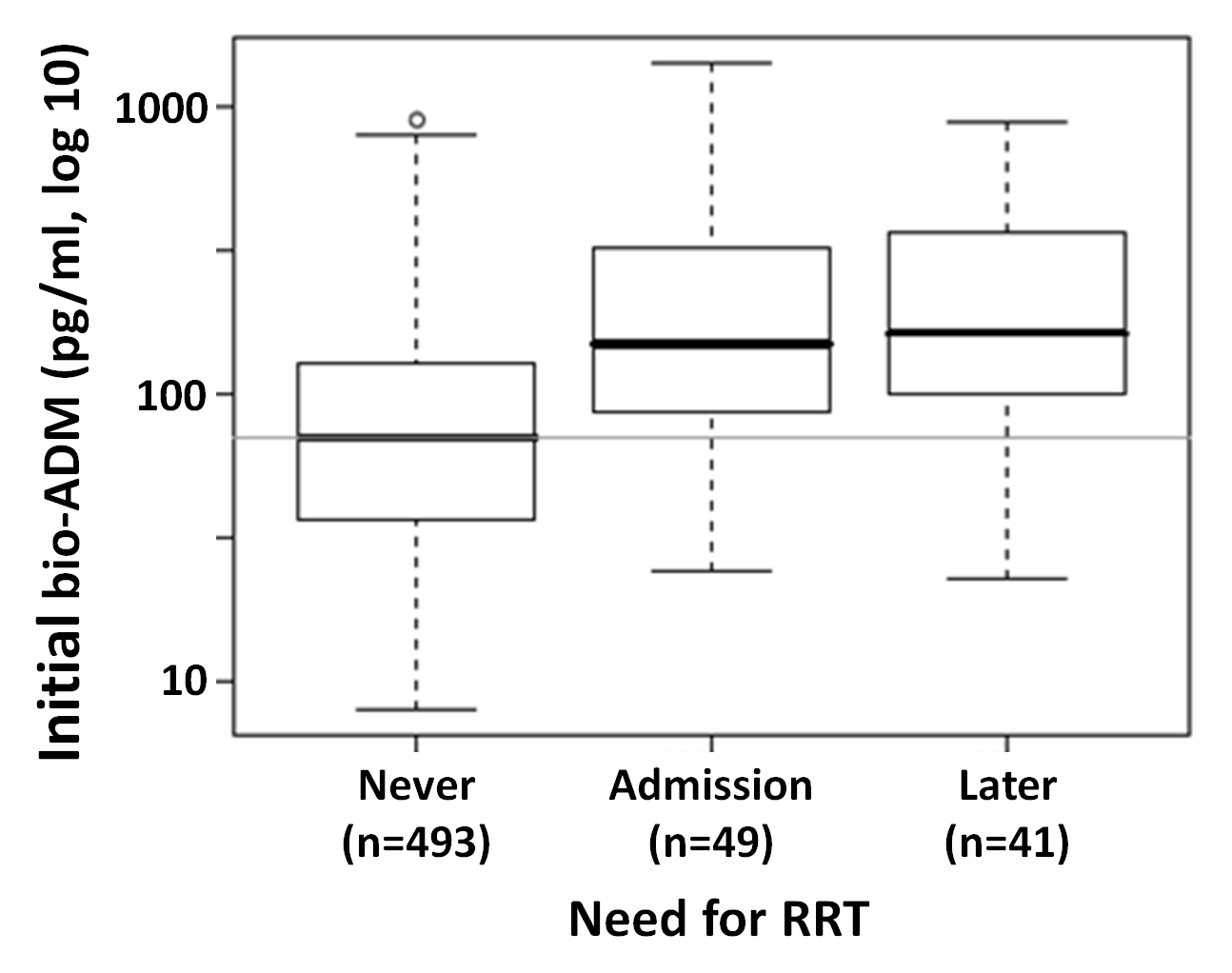

Supplement: Supplementary file 9 — Figure S6. Association between bio-ADM concentration on admission and need for renal replacement therapy on admission, later during ICU stay, or never (70.4 [36.3–128.8] vs. 149.0 [87.1–320.5] and 162.6 [99.8–367.3] pg/ml, for patients without need for RRT, on admission, or later during ICU stay, respectively, p < 0.0001). (TIF 221 kb) [file 13054_2018_2243_MOESM9_ESM.tif]

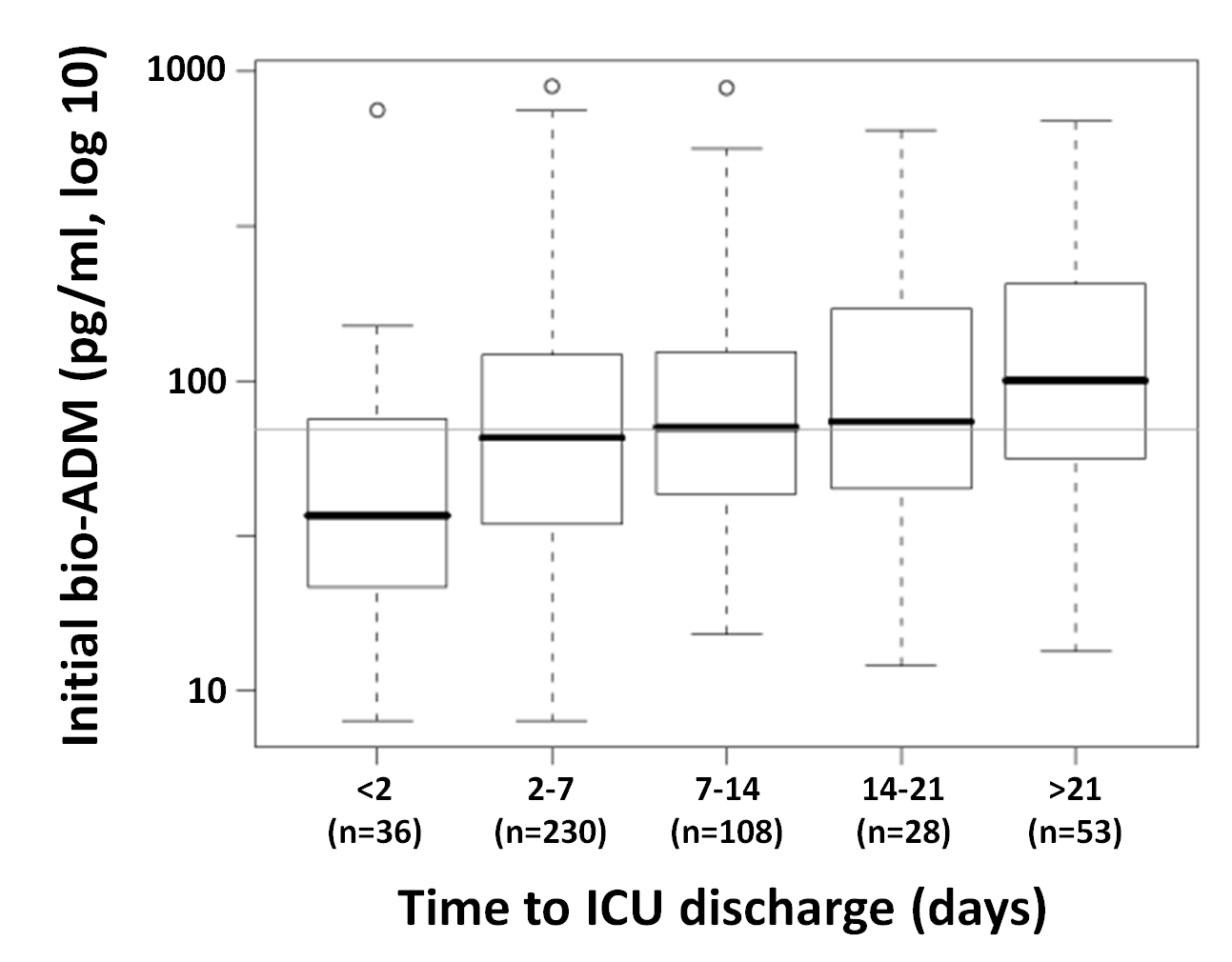

Supplement: Supplementary file 10 — Figure S7. Bio-ADM levels upon admission in 28-day survivors and time to ICU discharge (p < 0.0001): Patients with early discharge (< 2 days) are significantly different from all other groups (all p < 0.016), and late discharge (> 21 days) is significantly different from early discharge (< 2 days and 2–7 days, both p < 0.013). (TIF 217 kb) [file 13054_2018_2243_MOESM10_ESM.tif]

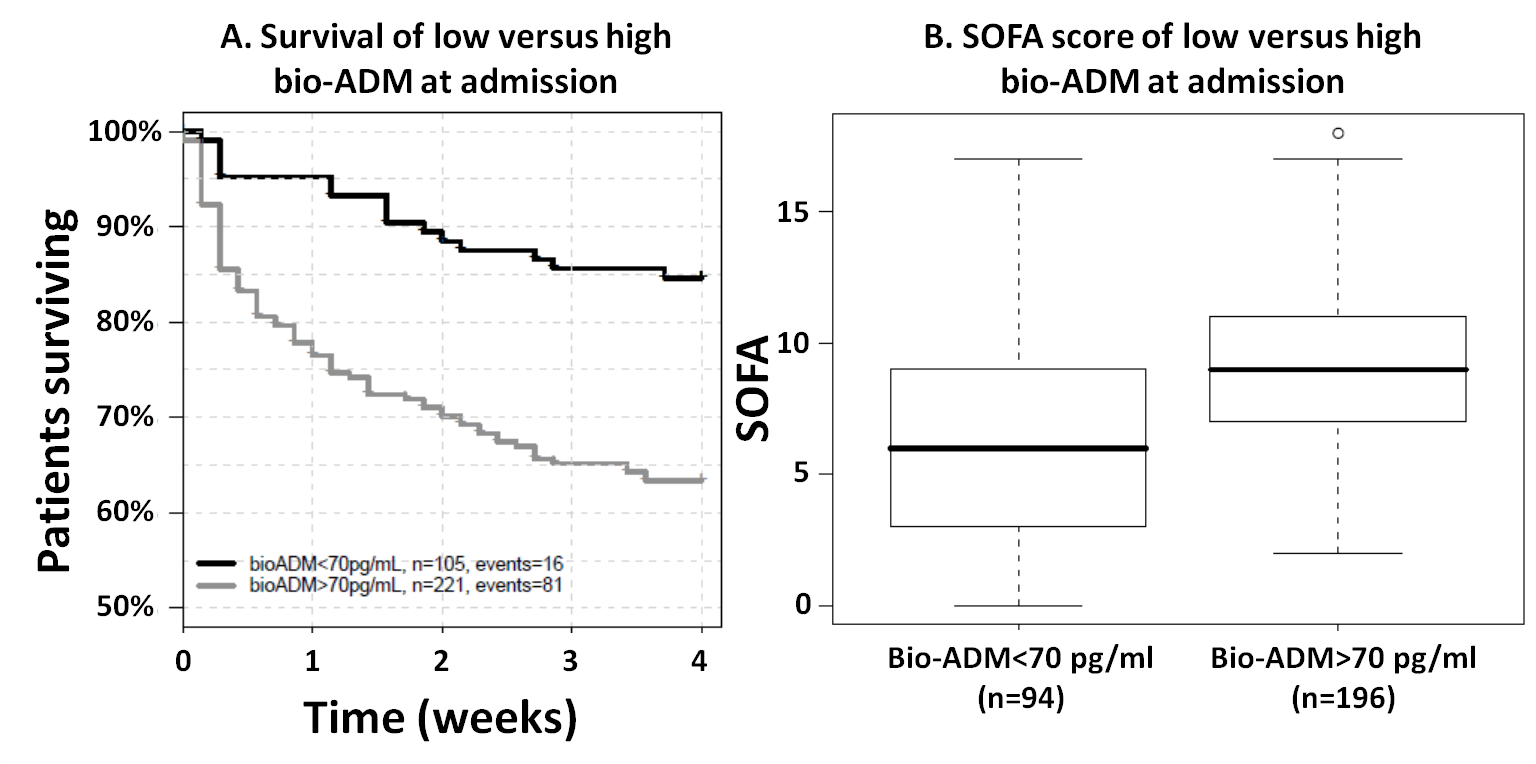

Supplement: Supplementary file 11 — Figure S8. Twenty-eight-day Kaplan-Meier survival curves of low versus high bio-ADM at admission, based on a cutoff value of 70 pg/ml, in patients with lactate > 2 mmol/L (p < 0.0001) (a) and SOFA score (b) for low versus high bio-ADM at admission (p < 0.0001). (TIF 229 kb) [file 13054_2018_2243_MOESM11_ESM.tif]
