# Supplementary material for: Circulating adrenomedullin estimates survival and reversibility of organ failure in sepsis: the prospective observational multinational Adrenomedullin and Outcome in Sepsis and Septic Shock-1 (AdrenOSS-1) study
Source: Crit Care. 2018 Dec 21;22:354. doi: 10.1186/s13054-018-2243-2 (PMC6305573; doi:10.1186/s13054-018-2243-2)
Supplement: Supplementary file 2 — Table S1. Patient characteristics of survivors and nonsurvivors. (DOCX 50 kb) [file 13054_2018_2243_MOESM2_ESM.docx]

**Supplementary Tables – AdrenOSS**

**Table S1.** Patient characteristics of survivors and non-survivors.

| **Patient characteristics** | **all** | **28-day survivors** | **28-day non-survivor** | **p-value*** | **n** |
| --- | --- | --- | --- | --- | --- |
| **Epidemiological data** | **n=583** | **n=456** | **n=127** |  |  |
| Bio-ADM at admission (pg/ml) | 80.5 [41.5-148.0] | 70.2 [35.8-127.4] | 131.0 [74.0-278.5] | <0.0001 |  |
| Age (year) | 66 [55-76] | 65 [54-74] | 70 [62-78] | 0.0001 |  |
| Males (n, %) | 364 (62.4) | 278 (61) | 86 (67.7) | 0.1985 |  |
| Body Mass Index (kg/m²) | 25.7 [22.9-30.1] | 25.75 [22.5-30.3] | 25.52 [23.7-29.8] | 0.5693 |  |
| Septic shock at admission | 293 (50.3) | 199 (43.6) | 94 (74) | <0.0001 |  |
| **Type of ICU admission:** |  |  |  | 0.5724 |  |
| Medical | 473 (81.1) | 374 (82) | 99 (78) |  |  |
| Surgical - emergency procedure | 93 (16) | 69 (15.1) | 24 (18.9) |  |  |
| Surgical - elective procedure | 17 (2.9) | 13 (2.9) | 4 (3.1) |  |  |
| **Origin of sepsis:** |  |  |  | 0.0408 |  |
| Lung | 218 (37.4) | 176 (38.6) | 42 (33.1) |  |  |
| Blood stream | 90 (15.4) | 81 (17.8) | 9 (7.1) |  |  |
| Urinary tract | 62 (10.6) | 43 (9.4) | 19 (15) |  |  |
| Catheter | 29 (5) | 23 (5) | 6 (4.7) |  |  |
| Peritonitis | 31 (5.3) | 24 (5.3) | 7 (5.5) |  |  |
| Endocarditis | 31 (5.3) | 21 (4.6) | 10 (7.9) |  |  |
| Bile duct infection | 8 (1.4) | 6 (1.3) | 2 (1.6) |  |  |
| CNS | 4 (0.7) | 3 (0.7) | 1 (0.8) |  |  |
| Skin and soft tissue | 10 (1.7) | 9 (2) | 1 (0.8) |  |  |
| Gynaecologic | 2 (0.3) | 2 (0.4) | 0 (0) |  |  |
| Other | 98 (16.8) | 68 (14.9) | 30 (23.6) |  |  |
| **Medical history**** |  |  |  |  |  |
| Any cardiac comorbidity | 400 (68.6) | 298 (65.4) | 102 (80.3) | 0.0019 |  |
| Chronic Heart Failure | 60 (10.3) | 43 (9.4) | 17 (13.4) | 0.2530 |  |
| Hypertension | 293 (50.3) | 214 (46.9) | 79 (62.2) | 0.0019 |  |
| Diabetes mellitus | 160 (27.4) | 120 (26.3) | 40 (31.5) | 0.2793 |  |
| Any non-cardiac comorbidity | 414 (71) | 309 (67.8) | 105 (82.7) | 0.0015 |  |
| Chronic renal disease | 76 (13.0) | 57 (12.5) | 19 (15.0) | 0.5586 |  |
| Active/recent malignant tumors | 124 (21.3) | 88 (19.3) | 36 (28.3) | 0.0272 |  |
| Smoking (active) | 117 (20.1) | 90 (19.7) | 27 (21.3) | 0.8099 |  |
| COPD | 89 (15.3) | 67 (14.7) | 22 (17.3) | 0.4932 |  |
| Any chronic medication | 371 (63.6) | 278 (61) | 93 (73.2) | 0.0148 |  |
| Immunosuppressive therapy | 46 (7.9) | 37 (8.1) | 9 (7.1) | 0.8464 |  |
| **Physiological values at admission** |  |  |  |  |  |
| Temperature (°C) | 37.2 [36.4-38.2] | 37.3 [36.6-38.2] | 36.8 [36-37.8] | 0.0010 |  |
| Mean blood pressure (mmHg) | 75 [64-90] | 76 [65-91] | 73 [60-90] | 0.1322 |  |
| Heart rate (bpm) | 104 [90-119] | 103 [88-117] | 110 [94-130] | 0.0077 |  |
| Central Venous pressure (mmHg) | 8 [5-13] | 8 [5-13] | 10 [6-13] | 0.4690 |  |
| Glasgow score | 15 [14-15] | 15 [14-15] | 15 [12-15] | <0.0001 |  |
| Fluid Balance (ml) | 1928 [592-3552] | 1745 [500-3131] | 2853.5 [1145-5036] | <0.0001 |  |
| Urine output for 24 hours (ml) | 1000 [450-1900] | 1158 [583-1996] | 550 [223-1440] | <0.0001 |  |
| PaO_2_/FiO_2_ | 228 [137-340] | 234 [146-354] | 190 [114-298] | 0.0059 |  |
| **Laboratory values at admission** |  |  |  |  |  |
| Lactate (mmol/l) | 1.4 [1.0-2.2] | 1.2 [0.9-1.9] | 2.2 [1.4-4.0] | <0.0001 | n=562 |
| Arterial pH | 7.38 [7.3-7.44] | 7.4 [7.32-7.45] | 7.33 [7.27-7.4] | <0.0001 |  |
| Bilirubin (umol/L) | 11 [6-19] | 10 [6-18] | 13 [7-24] | 0.0961 |  |
| Platelets (10^9^/L) | 190 [121-275] | 193 [124-281] | 166 [96-261] | 0.0301 |  |
| Creatinine (mg/dL) | 1.4 [0.9-2.2] | 1.3 [0.8-2.1] | 1.6 [1.1-2.6] | 0.0023 |  |
| BUN or Urea (mg/dL) | 61 [37-107] | 57 [34-101] | 75 [49-120] | 0.0002 |  |
| Hematocrit (%) | 34 [29-38] | 34 [30-38] | 33 [28-39] | 0.4325 |  |
| White blood count (per mm^3^) | 12525 [7200-18585] | 13345 [8000-18975] | 10365 [4150-16770] | 0.0008 |  |
| Troponin T, maximum on day 1 | 42 [18-158] | 33 [18-131] | 61 [34-241] | 0.0989 | n=153 |
| Troponin I, maximum on day 1 | 69 [20-246] | 60 [17-228] | 150 [39-1001] | 0.0468 | n=186 |
| PCT, maximum on day 1 (ng/mL) | 11.4 [1.9-49.8] | 10.7 [1.9-49.4] | 11.8 [2.2-52.5] | 0.6690 | n=330 |
| PCT, central lab (ng/mL) | 10.2 [2.3-34.3] | 9.7 [2.2-31.5] | 14.8 [3.9-42.8] | 0.0442 | n=583 |
| BNP, maximum on day 1 | 257 [102-723] | 239 [87.7-650] | 514 [139-1242] | 0.1183 | n=131 |
| NT-proBNP, maximum on day 1 | 4382 [1525-11565] | 3400.5 [1026.88-9052] | 7249 [4564-25007] | 0.0007 | n=117 |
| **Organ support at admission** |  |  |  |  |  |
| Mechanical ventilation: |  |  |  | <0.0001 |  |
| Invasive | 219 (37.6) | 141 (30.9) | 78 (61.4) |  |  |
| Non-invasive | 131 (22.5) | 107 (23.5) | 24 (18.9) |  |  |
| None | 233 (40.0) | 208 (45.6) | 25 (19.7) |  |  |
| Renal replacement therapy | 49 (8.4) | 27 (5.9) | 22 (17.3) | 0.0002 |  |
| Vasopressors/inotropes at admission | 346 (59.9) | 246 (53.9) | 103 (81.1) | <0.0001 |  |
| **Organ dysfunction scores** |  |  |  |  |  |
| SOFA (points) | 7 [5-10] | 6 [4-9] | 10 [7.5-12] | <0.0001 | n=509 |
| APACHE II (points) | 15 [11-20] | 15 [10-19] | 19 [16-23.5] | <0.0001 |  |
| **Length of stay (days)** |  |  |  |  |  |
| ICU | 5 [2-10] | 5 [2-10] | 4 [2-8] | 0.0147 |  |
| **Mortality** |  |  |  |  |  |
| 28-day, deaths (%) | 127 (21.8) | 0 (0) | 127 (100) | <0.0001 |  |
| 90-day, deaths (%) | 166 (28.5) | 39 (8.6) | 127 (100) | <0.0001 |  |

APACHE, acute physiology and chronic health evaluation; Bio-ADM, bioactive adrenomedullin; BNP, brain-derived natriuretic peptide; BUN, blood urea nitrogen; CNS, central nervous system; COPD, chronic obstructive pulmonary disease; ICU, intensive care unit; PCT, procalcitonin; SOFA, sequential organ failure assessment; NT-proBNP, N-terminal brain natriuretic peptide.

* p-value from non-parametric Kruskal-Wallis or Chi^2^ test, respectively.

** most common comorbidities reported individually.
