# Supplementary material for: Circulating adrenomedullin estimates survival and reversibility of organ failure in sepsis: the prospective observational multinational Adrenomedullin and Outcome in Sepsis and Septic Shock-1 (AdrenOSS-1) study
Source: Crit Care. 2018 Dec 21;22:354. doi: 10.1186/s13054-018-2243-2 (PMC6305573; doi:10.1186/s13054-018-2243-2)
Supplement: Supplementary file 4 — Table S2. Patient characteristics of the four different groups with respect to adrenomedullin trajectory over the first 48 h after study inclusion. (DOCX 56 kb) [file 13054_2018_2243_MOESM4_ESM.docx]

**Supplementary Tables – AdrenOSS**

**Table S2.** Patient characteristics of the four different groups with respect to adrenomedullin trajectory over the first 48 hours after study inclusion.

| **Patient characteristics** | **all** | **Bio-ADM <70 pg/mL, admission and 48h** | **Bio-ADM <70 pg/mL on admission, but >70 pg/mL at 48h** | **Bio-ADM >70 pg/mL on admission, but <70 pg/mL at 48h** | **Bio-ADM >70 pg/mL, admission and 48h** | **p-value*** | **n** |
| --- | --- | --- | --- | --- | --- | --- | --- |
| **Epidemiological data** | **n=583** | **n=241 (41.3%)** | **n=16 (2.7%)** | **n=95 (16.3%)** | **n=231 (39.6%)** |  |  |
| Bio-ADM at admission (pg/ml) | 80.5 [41.5-148.0] | 35.7 [27.0-48.1] | 60.3 [55.8-66.0] | 95.4 [82.8-120.6] | 162.6 [118.0-301.0] | <0.0001 |  |
| Age (year) | 66 [55-76] | 64 [53-75] | 64 [55-70] | 67 [59-77] | 68 [58-76] | 0.0454 |  |
| Males (n, %) | 364 (62.4) | 159 (66) | 12 (75) | 60 (63.2) | 133 (57.6) | 0.1942 |  |
| Body Mass Index (kg/m^2^) | 25.7 [22.9-30.1] | 25.0 [22.5-28.4] | 25.1 [21.2-27.9] | 25.9 [22.5-30.7] | 26.9 [23.4-32.0] | 0.0048 |  |
| Septic shock at admission | 293 (50.3) | 75 (31.1) | 9 (56.2) | 52 (54.7) | 157 (68) | <0.0001 |  |
| **Type of ICU admission:** |  |  |  |  |  | 0.0007 |  |
| Medical | 473 (81.1) | 216 (89.6) | 14 (87.5) | 69 (72.6) | 174 (75.3) |  |  |
| Surgical - emergency procedure | 93 (16) | 20 (8.3) | 1 (6.2) | 22 (23.2) | 50 (21.6) |  |  |
| Surgical - elective procedure | 17 (2.9) | 5 (2.1) | 1 (6.2) | 4 (4.2) | 7 (3) |  |  |
| **Origin of sepsis:** |  |  |  |  |  | <0.0001 |  |
| Lung | 218 (37.4) | 123 (51) | 6 (37.5) | 30 (31.6) | 59 (25.5) |  |  |
| Blood stream | 90 (15.4) | 31 (12.9) | 0 (0) | 23 (24.2) | 36 (15.6) |  |  |
| Urinary tract | 62 (10.6) | 8 (3.3) | 2 (12.5) | 13 (13.7) | 39 (16.9) |  |  |
| Catheter | 29 (5) | 9 (3.7) | 0 (0) | 8 (8.4) | 12 (5.2) |  |  |
| Peritonitis | 31 (5.3) | 11 (4.6) | 1 (6.2) | 2 (2.1) | 17 (7.4) |  |  |
| Endocarditis | 31 (5.3) | 11 (4.6) | 1 (6.2) | 6 (6.3) | 13 (5.6) |  |  |
| Bile duct infection | 8 (1.4) | 2 (0.8) | 0 (0) | 2 (2.1) | 4 (1.7) |  |  |
| CNS | 4 (0.7) | 4 (1.7) | 0 (0) | 0 (0) | 0 (0) |  |  |
| Skin and soft tissue | 10 (1.7) | 9 (3.7) | 0 (0) | 1 (1.1) | 0 (0) |  |  |
| Gynaecologic | 2 (0.3) | 1 (0.4) | 0 (0) | 0 (0) | 1 (0.4) |  |  |
| Other | 98 (16.8) | 32 (13.3) | 6 (37.5) | 10 (10.5) | 50 (21.6) |  |  |
| **Medical history**** |  |  |  |  |  |  |  |
| Any cardiac comorbidity | 400 (68.6) | 135 (56) | 12 (75) | 71 (74.7) | 182 (78.8) | <0.0001 |  |
| Chronic Heart Failure | 60 (10.3) | 16 (6.6) | 3 (18.8) | 10 (10.5) | 31 (13.4) | 0.0697 |  |
| Hypertension | 293 (50.3) | 97 (40.2) | 8 (50.0) | 53 (55.8) | 135 (58.4) | 0.0004 |  |
| Diabetes mellitus | 160 (27.4) | 52 (21.6) | 5 (31.2) | 22 (23.2) | 81 (35.1) | 0.0075 |  |
| Any non-cardiac comorbidity | 414 (71) | 153 (63.5) | 14 (87.5) | 70 (73.7) | 177 (76.6) | 0.0056 |  |
| Chronic renal disease | 76 (13.0) | 18 (7.5) | 1 (6.2) | 13 (13.7) | 44 (19.0) | 0.0019 |  |
| Active/recent malignant tumor | 124 (21.3) | 28 (11.6) | 6 (37.5) | 26 (27.4) | 64 (27.7) | <0.0001 |  |
| Smoking (active) | 117 (20.1) | 60 (24.9) | 3 (18.8) | 15 (15.8) | 39 (16.9) | 0.1286 |  |
| COPD | 89 (15.3) | 34 (14.1) | 3 (18.8) | 19 (20.0) | 33 (14.3) | 0.5181 |  |
| Any chronic medication | 371 (63.6) | 126 (52.3) | 12 (75) | 62 (65.3) | 171 (74) | <0.0001 |  |
| Immunosuppressive therapy | 46 (7.9) | 10 (4.1) | 1 (6.2) | 7 (7.4) | 28 (12.1) | 0.0153 |  |
| **Physiological values at admission** |  |  |  |  |  |  |  |
| Temperature (°C) | 37.2 [36.4-38.2] | 37.3 [36.6-38.3] | 37.65 [36.8-38] | 37.1 [36.3-38] | 37.1 [36.2-38.1] | 0.0320 |  |
| Mean blood pressure (mmHg) | 75 [64-90] | 81 [69-97] | 77 [70-84] | 73 [61-87] | 71 [60-85] | <0.0001 |  |
| Heart rate (bpm) | 104 [90-119] | 99 [86-115] | 106 [92-127] | 104 [95-121] | 107 [94-122] | 0.0086 |  |
| Central Venous pressure (mmHg) | 8 [5-13] | 7.5 [5-13] | 9 [8-11] | 11 [6-14] | 10 [6-13] | 0.6906 |  |
| Glasgow score | 15 [14-15] | 15 [14-15] | 15 [15-15] | 15 [14-15] | 15 [14-15] | 0.2926 |  |
| Fluid Balance (ml) | 1928 [592-3552] | 1366 [483-2673] | 1973 [625-2821] | 1930 [730-3196] | 2629 [813-4798] | 0.0000 |  |
| Urine output for 24 h (ml) | 1000 [450-1900] | 1300 [700-2110] | 540 [285-1105] | 1095 [581-1947] | 630 [237-1540] | <0.0001 |  |
| PaO_2_/FiO_2_ | 228 [137-340] | 235 [143-368] | 204 [107-269] | 237 [155-365] | 209 [133-330] | 0.3173 |  |
| **Laboratory values at admission** |  |  |  |  |  |  |  |
| Lactate (mmol/l) | 1.4 [1.0-2.2] | 1.1 [0.8-1.6] | 1.3 [1.0-2.2] | 1.4 [1.0-2.0] | 2.0 [1.2-3.1] | <0.0001 | n=562 |
| Arterial pH | 7.38 [7.3-7.44] | 7.42 [7.36-7.46] | 7.38 [7.32-7.44] | 7.37 [7.32-7.44] | 7.35 [7.26-7.41] | <0.0001 |  |
| Bilirubin (umol/L) | 11 [6-19] | 10 [6-17] | 10.5 [7-17] | 11.5 [6-22] | 12 [6-21] | 0.5267 |  |
| Platelets (10^9^/L) | 190 [121-275] | 196 [136-275] | 191.5 [137-328] | 186 [116-262] | 178 [103-280] | 0.2895 |  |
| Creatinine (mg/dL) | 1.4 [0.9-2.2] | 1 [0.7-1.4] | 1.1 [0.8-1.8] | 1.8 [1.2-2.6] | 1.8 [1.2-3] | <0.0001 |  |
| BUN or Urea (mg/dL) | 61 [37-107] | 43 [28-67] | 52 [33-90] | 72 [46-121] | 82 [53-129] | <0.0001 |  |
| Hematocrit (%) | 34 [29-38] | 35 [30-38] | 33.5 [31.5-35.75] | 35 [30-38] | 33 [28-38] | 0.1831 |  |
| White blood count (per mm^3^) | 12525 [7200-18585] | 13210 [8507-17932] | 11995 [7457-20062] | 12375 [7692-18825] | 11445 [5427-19075] | 0.1783 |  |
| Troponin T, max on day 1 | 42 [18-158] | 27 [14-64] | 129 [33-554] | 37 [23-145] | 69 [26-187] | 0.0082 | n=153 |
| Troponin I, max on day 1 | 69 [20-246] | 45 [11-228] | 30 [25-762] | 91 [40-330] | 100 [40-254] | 0.0439 | n=186 |
| PCT, max on day 1 (ng/mL) | 11.4 [1.9-49.8] | 3.8 [0.9-19.7] | 4.6 [0.9-6.5] | 25.9 [7.7-70.7] | 19.3 [4.5-87] | <0.0001 | n=330 |
| PCT, max on day 1 (ng/mL) | 10.2 [2.3-34.3 | 3.8 [0.8-13.0] | 2.6 [1.3-10.1] | 17.4 [5.0-50.9] | 18.8 [6.3-53.9] | <0.0001 | n=583 |
| BNP, max on day 1 | 257 [102-723 | 185 [59-383] | 806 [428-592] | 372 [140-503] | 545 [166-1282] | 0.0007 | n=131 |
| NT-proBNP, max on day 1 | 4382 [1525-11565] | 2072 [459-5726] | 8691 [2301-17225] | 4970 [1951-11896] | 6229 [3560-18486] | 0.0001 | n=117 |
| **Organ support at admission** |  |  |  |  |  |  |  |
| Mechanical ventilation: |  |  |  |  |  | 0.0004 |  |
| Invasive | 219 (37.6) | 76 (31.5) | 9 (56.2) | 24 (25.3) | 110 (47.6) |  |  |
| Non-invasive | 131 (22.5) | 63 (26.1) | 4 (25) | 22 (23.2) | 42 (18.2) |  |  |
| None | 233 (40.0) | 102 (42.3) | 3 (18.8) | 49 (51.6) | 79 (34.2) |  |  |
| Renal replacement therapy | 49 (8.4) | 8 (3.3) | 0 (0) | 7 (7.4) | 34 (14.7) | 0.0008 |  |
| Vasopressors/inotropes at admission | 349 (59.9) | 98 (40.7) | 11 (68.8) | 59 (62.1) | 181 (78.4) | <0.0001 |  |
| **Organ dysfunction scores** |  |  |  |  |  |  |  |
| SOFA (points) | 7 [5-10] | 5 [3-8] | 7.5 [6-10] | 7 [5-9] | 9 [7-11] | <0.0001 | n=509 |
| APACHE II (points) | 15 [11-20] | 14 [9-17] | 15.5 [13-19.5] | 16 [12.5-20] | 18 [14-22.5] | <0.0001 |  |
| **Length of stay (days)** |  |  |  |  |  |  |  |
| ICU | 5 [2-10] | 4 [2-8] | 10 [7.5-15.5] | 5 [3-7.5] | 5 [2-13] | 0.0002 |  |
| **Mortality** |  |  |  |  |  |  |  |
| 28-day, deaths (%) | 127 (21.8) | 25 (10.4) | 5 (31.2) | 9 (9.5) | 88 (38.1) | <0.0001 |  |
| 90-day, deaths (%) | 166 (28.5) | 36 (14.9) | 5 (31.2) | 16 (16.8) | 109 (47.2) | <0.0001 |  |

APACHE, acute physiology and chronic health evaluation; Bio-ADM, bioactive adrenomedullin; BNP, brain-derived natriuretic peptide; BUN, blood urea nitrogen; CNS, central nervous system; COPD, chronic obstructive pulmonary disease; ICU, intensive care unit; PCT, procalcitonin; SOFA, sequential organ failure assessment; NT-proBNP, N-terminal brain natriuretic peptide.

* p-value from non-parametric Kruskal-Wallis or Chi^2^ test, respectively. ** most common comorbidities reported individually.
