# Supplementary material for: Circulating adrenomedullin estimates survival and reversibility of organ failure in sepsis: the prospective observational multinational Adrenomedullin and Outcome in Sepsis and Septic Shock-1 (AdrenOSS-1) study
Source: Crit Care. 2018 Dec 21;22:354. doi: 10.1186/s13054-018-2243-2 (PMC6305573; doi:10.1186/s13054-018-2243-2)
Supplement: Supplementary file 8 — Table S3. Association between adrenomedullin and need of vasopressors/inotropes at admission. (DOCX 23 kb) [file 13054_2018_2243_MOESM8_ESM.docx]

**Table S3.** Association between adrenomedullin and need of vasopressors/inotropes at admission*.*

|  | **All** | **Bio-ADM <70 pg/mL** | **Bio-ADM >70 pg/mL** | **p-value** |
| --- | --- | --- | --- | --- |
| Dobutamine (%) | 23 (4) | 9 (4) | 14 (4) | 0.7842 |
| Dobutamine (%) | 23 (4) | 9 (4) | 14 (4) | 0.7842 |
| Epinephrine (%) | 16 (3) | 3 (1) | 13 (4) | 0.0696 |
| Norephinephrine (%) | 346 (59) | 107 (42) | 239 (73) | <0.0001 |
| Dose of norepinephrine  (μg/kg/min; median [IQR]) | 0.3 [0.2-0.7] | 0.2 [0.1-0.4] | 0.4 [0.3-0.8] | 0.0022 |

Data are expressed as median [IQR, interquartile range] or as number of patients (percentage). Bio-ADM, bioactive adrenomedullin.
