# Supplementary material for: Circulating adrenomedullin estimates survival and reversibility of organ failure in sepsis: the prospective observational multinational Adrenomedullin and Outcome in Sepsis and Septic Shock-1 (AdrenOSS-1) study
Source: Crit Care. 2018 Dec 21;22:354. doi: 10.1186/s13054-018-2243-2 (PMC6305573; doi:10.1186/s13054-018-2243-2)
Supplement: Supplementary file 12 — Table S4. Comparison of AdrenOSS-1 and ALBIOS. (DOCX 24 kb) [file 13054_2018_2243_MOESM12_ESM.docx]

**Table S4.** Comparison of AdrenOSS-1 and ALBIOS*.*

|  | **AdrenOSS-1**  **n = 583** | **ALBIOS**  **n = 956** |
| --- | --- | --- |
| Definition of septic shock | Sepsis-2 [1] | SOFA score 3-4 |
| Septic shock patients (%) | 50.3 | 56.4 |
| Baseline Bio-ADM value (median, pg/mL) | 80.5 | 110 |
| Bio-ADM value in severe sepsis (median, pg/mL) | 58 | 86 |
| Bio-ADM value in septic shock (median, pg/mL) | 114 | 122 |
| Mechanical ventilation upon study inclusion (%) | 60 | 82.5 |
| Length of ICU stay (median, IQR) | 5 [2-10] | 10 [5-20] |
| 90-day mortality (%) | 29 | 39 |

Data are expressed as time range, median [IQR, interquartile range] or as number of patients (percentage). Bio-ADM, bioactive adrenomedullin.

**Reference**

1. Levy MM, Fink MP, Marshall JC, Abraham E, Angus D, Cook D, Cohen J, Opal SM, Vincent JL, Ramsay G *et al*: **2001 SCCM/ESICM/ACCP/ATS/SIS International Sepsis Definitions Conference**. *Crit Care Med* 2003, **31**(4):1250-1256.
